# Discovery of 2-(((1*r*,4*r*)-4-(((4-Chlorophenyl)(phenyl)carbamoyl)oxy)methyl)cyclohexyl)methoxy)acetate (Ralinepag): An Orally Active Prostacyclin Receptor Agonist for the Treatment of Pulmonary Arterial Hypertension

**DOI:** 10.1021/acs.jmedchem.6b00871

**Published:** 2017-01-10

**Authors:** Thuy-Anh Tran, Bryan Kramer, Young-Jun Shin, Pureza Vallar, P. Douglas Boatman, Ning Zou, Carleton R. Sage, Tawfik Gharbaoui, Ashwin Krishnan, Biman Pal, Sagar R. Shakya, Antonio Garrido Montalban, John W. Adams, Juan Ramirez, Dominic P. Behan, Anna Shifrina, Anthony Blackburn, Tina Leakakos, Yunqing Shi, Michael Morgan, Abu Sadeque, Weichao Chen, David J. Unett, Ibragim Gaidarov, Xiaohua Chen, Steve Chang, Hsin-Hui Shu, Shiu-Feng Tung, Graeme Semple

**Affiliations:** Arena Pharmaceuticals, 6154 Nancy Ridge Drive, San Diego, California 92121, United States

## Abstract

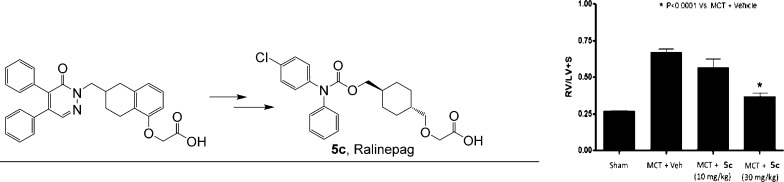

The design and synthesis of a new series of potent non-prostanoid IP receptor agonists that showed oral efficacy in the rat monocrotaline model of pulmonary arterial hypertension (PAH) are described. Detailed profiling of a number of analogues resulted in the identification of **5c** (ralinepag) that has good selectivity in both binding and functional assays with respect to most members of the prostanoid receptor family and a more modest 30- to 50-fold selectivity over the EP3 receptor. In our hands, its potency and efficacy are comparable or superior to MRE269 (the active metabolite of the clinical compound NS-304) with respect to in vitro IP receptor dependent cAMP accumulation assays. **5c** had an excellent PK profile across species. Enterohepatic recirculation most probably contributes to a concentration–time profile after oral administration in the cynomolgus monkey that showed a very low peak-to-trough ratio. Following the identification of an acceptable solid form, **5c** was selected for further development for the treatment of PAH.

## Introduction

**Figure 1:**
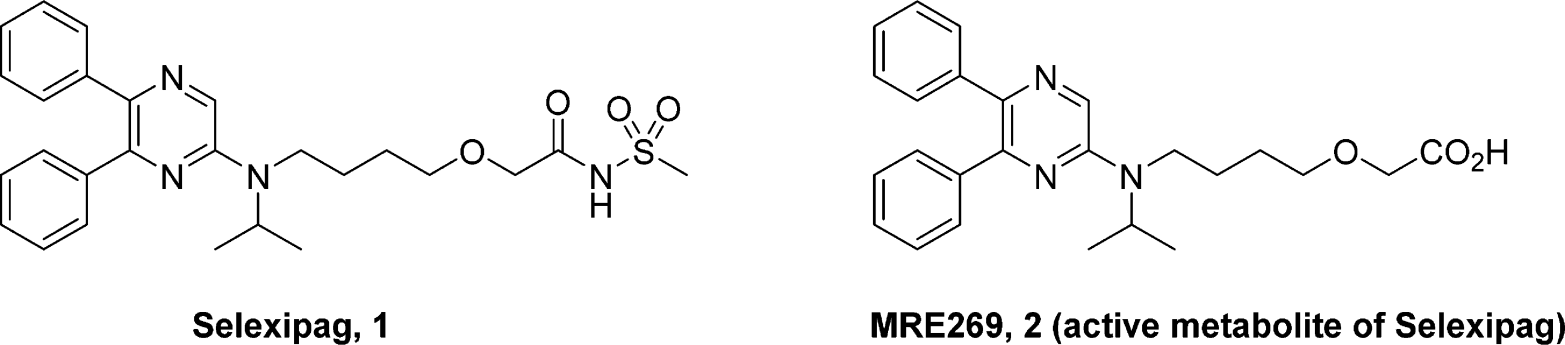
Structures of selexipag and its active metabolite.

Pulmonary arterial hypertension (PAH) is a rare but life-threatening disease. The pathogenesis, which is thought to result in part from an imbalance in the production of two cyclooxygenase metabolites of arachidonic acid, prostacyclin (PGI2) and thromboxane A2,^1,2^ includes pulmonary vasoconstriction, endothelial and smooth muscle cell proliferation, tissue remodeling, and platelet mediated thrombosis.^3^ The protective effects of PGI2 arise via activation of the prostacyclin receptor (IP) which leads to stimulation of adenylate cyclase, with a resulting increase in intracellular cAMP levels, in platelets, smooth muscle cells, and immune cells. Therapeutic substitution of PGI2 or its analogues in PAH patients can reduce pulmonary artery pressure and slow disease progression.^4^ This class of compounds generally has short half-lives in vivo due to chemical and metabolic instability and has to be dosed by repeated inhalation or continuous intravenous infusion. As a result, many of these drugs have limited and sometimes problematic clinical application. Other drug classes have also been investigated for this indication, and several orally available endothelin antagonists (that act by blocking the vasoconstrictive effects of endothelin peptides) and PDE5 inhibitors (that act by blocking the degradation of cGMP which is vasodilatory) have been approved creating a multibillion dollar market and an improvement in patient care in this disease area.^5^ To improve upon the currently approved prostacyclin based drugs and to be more competitive with the other classes in terms of compliance, stable, orally bioavailable PGI2 analogs and non-prostanoid IP receptor agonists have been investigated.^6^ One such compound, NS-304 or selexipag, **1** which is a prodrug of the active species MRE269 (**2**, Figure 1),^7^ was recently approved for the treatment of PAH.

The larger prostanoid ligand family evokes diverse biological actions in many tissues and cell types by binding to multiple specific cell surface G-protein-coupled receptors including prostaglandin D_2_ receptors (DP1, DP2), prostaglandin E_2_ receptors (EP1, EP2, EP3, EP4), prostaglandin F_2a_ receptor (FP), and the thromboxane receptor (TP) in addition to IP. Thus, we sought to design new prostacyclin receptor agonists with significantly improved pharmacokinetic profiles that, like selexipag, might be dosed orally but that would not require metabolic activation while staying mindful of the requirement for good receptor selectivity to avoid other prostaglandin mediated activities. Although the majority of clinical side effects of current prostacyclin agonists are thought to be on target, activation of other prostaglandin receptors, such as EP3 in the gut for example, may contribute to some of the GI symptoms observed with previous agents.^7^

## Results and Discussion

### Designing and Synthesis of a New Series of IP Agonists

**Figure 2:**
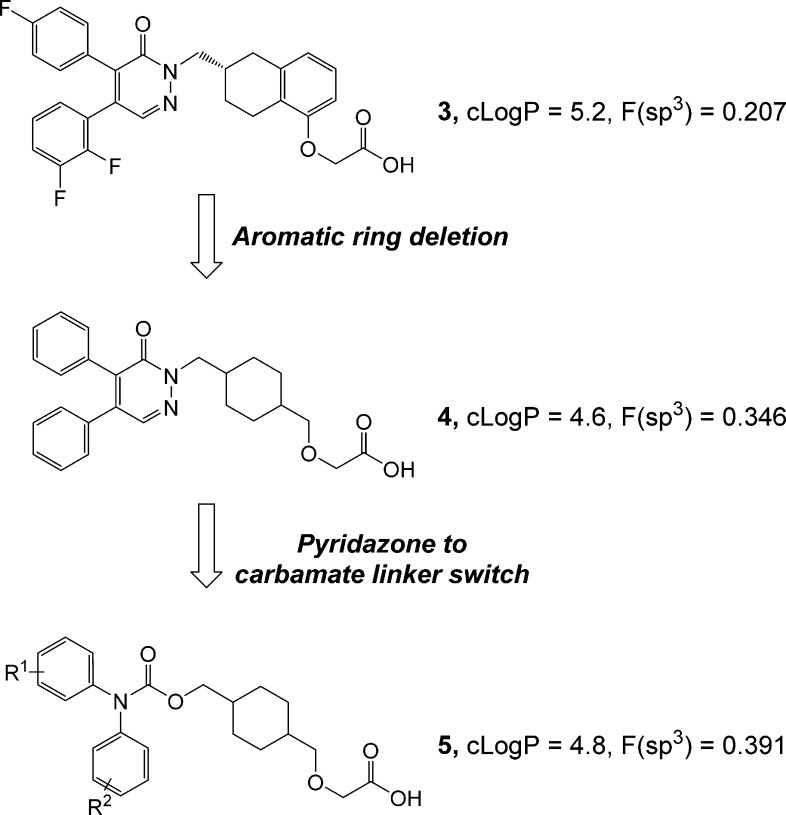
Simplification approach to redesign of prostacyclin agonist series.

For our earlier approaches to new IP receptor agonists, we surveyed multiple series of compounds designed from an understanding of the structural requirements for activity based on previously known non-prostanoid IP receptor agonists.^8^ Compounds of this type were shown to be potent agonists of the IP receptor with good selectivity over most prostanoid receptor family members but only very modest selectivity over the DP1 receptor. **3** (Figure 2) was optimal in this series but had an IP to DP1 selectivity ratio of just 8-fold. Analogues from this series did demonstrate in vivo activity after sc dosing in the widely used rat monocrotaline model of PAH.^9^ However, this early design process failed to provide compounds with significant oral activity in the same model. As a result, our redesign of these initial prostacyclin agonist series focused on three key objectives. The first was to improve selectivity over DP1. Second, we aimed to reduce aromatic ring count and lipophilicity in an effort to improve the in vivo, and eventually the clinical, profile. It has been suggested that either an increase in the sp^3^ character^10^ or the reduction in the number of aromatic rings in candidate drugs may correlate with improved clinical success.^11^ Finally, we were interested in significantly reducing the cycle times for synthesis and testing by trying to target compounds with a shorter synthetic route compared to the 10–14 step syntheses required to prepare our first series. We achieved the second of these objectives by deleting the fused aromatic ring in our first series (Figure 2; *F*(sp^3^) was increased by 2-fold, albeit without any significant change in clogP). This approach had the added advantage of removing a chiral center which helped to simplify the synthesis, although two alternative relative stereochemical arrangements are possible. In addition, as this type of scaffold was new in the prostaglandin space, it also opened up a number of additional possibilities for the linker group that serves to orientate the two required aromatic groups, giving us a further opportunity to reduce synthetic complexity. After briefly investigating a number of such linker–aromatic combinations, including using both of the pyridazinone building blocks as well as some related heterocycles we explored previously, we quickly settled on the carbamate series **5**, which was relatively simple to prepare in only a few steps.

**Scheme 1:**
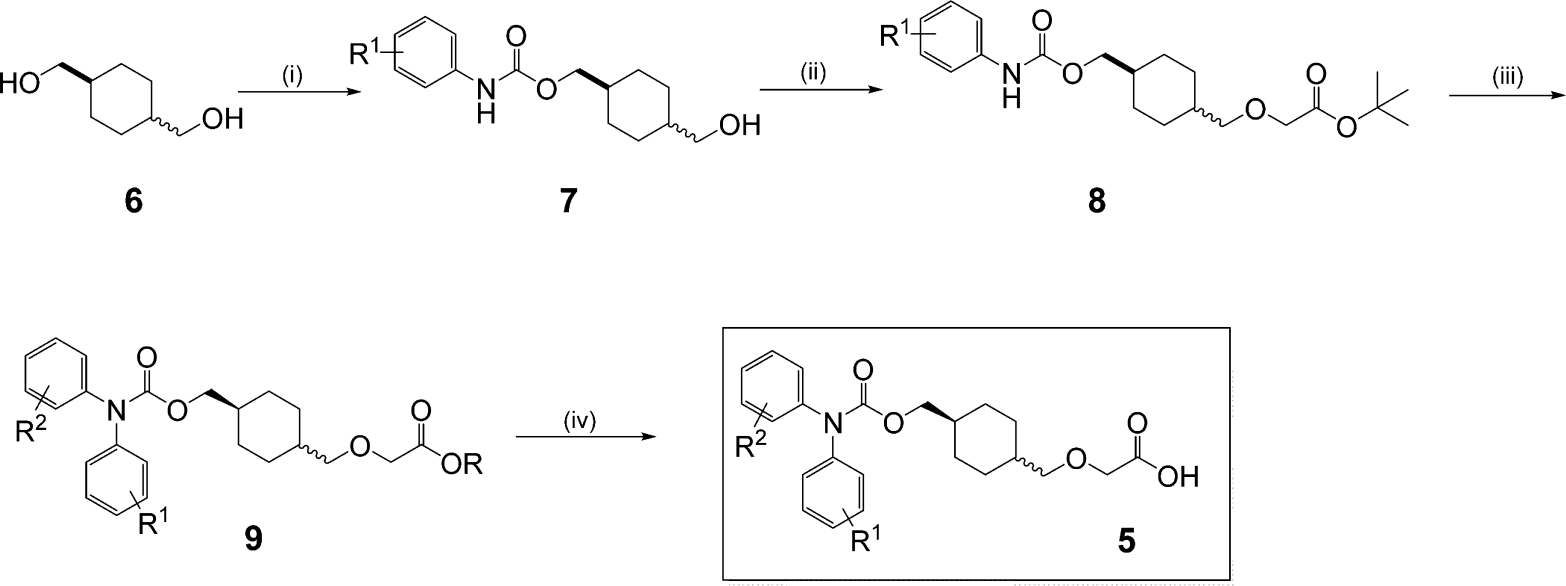
Library Route for the Preparation of the Cyclohexyl Scaffold/Carbamate Series Reagents and conditions: (i) R^1^PhNCO, pyridine; (ii) *tert*-butyl diazoacetate, (Rh(OAc)_2_)_2_; (iii) R^2^I, CuI, K_3_PO_4_, microwave; (iv) HCl, dioxane.

For our initial investigation, we prepared both the *cis-* and *trans-*analogues for a number of biaryl carbamates by using either the *cis-* or *trans-*cyclohexanedimethanol building block (**6**, Scheme 1) and reacting it with the appropriate isocyanate (e.g., R_1_ = H) to provide **7**. The alcohol was then elongated using a rhodium acetate catalyzed insertion reaction with *tert*-butyl diazoacetate to provide the *tert*-butyl ester **8** in good yield. The target compounds were then prepared in parallel format by direct Ullman-like arylation of the carbamate to give **9** followed by simple acid catalyzed deprotection of the ester group to provide the desired compounds **5**.

### In Vitro SAR of New Series

The new compounds were tested for functional agonist activity in a standard (Cisbio) cAMP assay using recombinant IP or DP1 receptors stably expressed in CHO-K1 cells. Clonal cell lines were derived following standard protocols, but receptor expression levels were kept to a minimum to preclude receptor reserve effects. To streamline our screening process, we focused on the DP1 receptor as the most likely off-target issue based on the experience with our earlier series as discussed above and that the new compounds had been designed using modifications to that series. In addition, the first compound in the series **5a** showed no activity at prostaglandin receptors other than DP1 based on a panel assays in melanophores. More extensive profiling with other family receptors was therefore performed only on potential preclinical candidate compounds. This approach was later shown to be flawed when we noted some previously unseen off-target activity with compounds that were more potent than **5a** on the IP receptor. Our new compounds were compared to **2** (the active metabolite of the approved compound **1**(12)) for receptor potency and intrinsic activity at the IP receptor, as we felt this to be the most appropriate comparator compound for our program, as at the time **1** was the only orally acting non-prostanoid IP agonist drug in development. In all cases (Table 1) the *trans*-relative stereochemical arrangement provided compounds with significantly greater potency in the human IP receptor cAMP assay than their *cis*-counterparts (**5a**–**h**), with the biggest difference being observed between the 4-methoxy analogues **5e** and **5f**. From this point onward, only the *trans*-isomers were prepared as we attempted to further optimize the series.

**Table 1:**
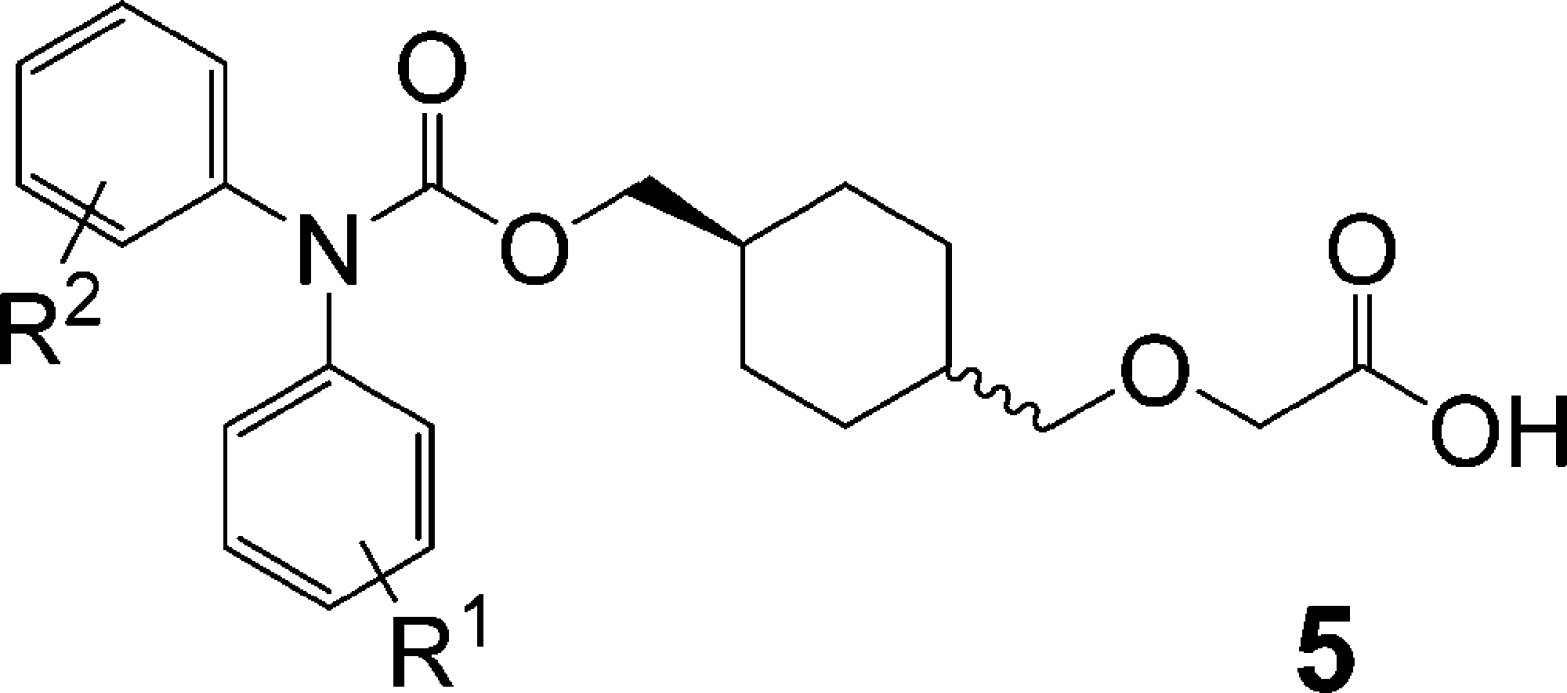
Screening Data for Selected Analogues^a^

	rel conf	R_1_	R_2_	EC_50_ hIP,^b^nM (% IA)	EC_50_ rIP,^c^nM (% IA)	EC_50_ hDP1,^d^nM (% IA^e^)	human platelet, IC_50_ (nM)^f^	human and rat MS, *T*_1/2_ (min)^g^
**1**				6 (98)	200 (100)	100 (48)	62	h, >60; r, >60
**5a**	*trans*	H	H	21 (77)	365 (89)	355 (59)	25	h, >60; r, >60
**5b**	*cis*	H	H	54 (81)	472 (94)	1710 (68)	54	nd
**5c**	*trans*	H	4-Cl	8.5 (85)	530 (88)	850 (52)	38	h, >60; r, >60
**5d**	*cis*	H	4-Cl	46 (80)	560 (88)	980 (60)	166	nd
**5e**	*trans*	H	4-OMe	3.3 (83)	165 (91)	240 (74)	22	h, >60; r, >60
**5f**	*cis*	H	4-OMe	1630 (16)	>10000	nd	nd	nd
**5g**	*trans*	H	3-F	8.7 (85)	284 (83)	2760 (61)	18	h, >60; r, >60
**5h**	*cis*	H	3-F	62 (79)	561 (88)	3870 (61)	93	nd
**5i**	*trans*	H	3-Cl	19 (80)	272 (88)	5070 (30)	54	h, >60; r, >60
**5j**	*trans*	H	3-OMe	12 (92)	470 (94)	1720 (68)	23	h, >60; r, 53
**5k**	*trans*	H	4-Me	3.9 (81)	174 (99)	320 (53)	39	nd
**5l**	*trans*	H	4-F	6.6 (89)	535 (91)	620 (55)	12	h, >60; r, >60
**5m**	*trans*	H	4-Cl, 3-F	3.4 (85)	400 (92)	2050 (38)	23	h, >60; r, >60
**5n**	*trans*	H	3-Cl, 4-F	38 (79)	1330 (102)	>10000	142	h, >60; r, >60
**5o**	*trans*	H	3,4-F_2_	10 (88)	640 (95)	5000 (45)	19	h, >60; r, >60
**5p**	*trans*	4-OMe	3-F	5.2 (70)	1570 (60)	1030 (65)	43	nd
**5q**	*trans*	4-Cl	3-F	18.5 (70)	3190 (44)	1140 (35)	nd	h, >60; r, >60
**5r**	*trans*	4-F	4-F	41 (65)	>10000	860 (45)	nd	nd
**5s**	*trans*	4-F	3-F	7.8 (76)	1630 (54)	808 (57)	30	h, >60; r, >60
**5t**	*trans*	3-F	4-Cl, 3-F	23 (75)	2720 (56)	>10000	nd	h, >60; r, >60
**2**				22 (60)	nd	154 (80)	288	nd
iloprost				2.4 (101)	0.69 (99)	147 (42)	9	nd

a nd = not determined. rel conf = relative stereochemical configuration of the 1,4-substituents around the cyclohexyl ring core.

b EC_50_ in the HTRF (cAMP) human or rat IP receptor assay. Data are the mean of at least three determinations with log(SD) < 0.33.

c Intrinsic activity (efficacy) relative to 1 μM iloprost as the positive control.

d EC_50_ in the HTRF (cAMP) human DP1 receptor assay. Data are the mean of at least three determinations with log(SD) < 0.4.

e Intrinsic activity (efficacy) relative to 1 μM PGD_2_ as the positive control.

f Inhibition of ADP-induced human platelet aggregation. Data are the mean of at least two determinations. All compounds with a measurable IC_50_ were able to fully inhibit the platelet aggregation induced by ADP.

g Half-life after incubation at 37 °C in human and rat microsome preparations.

The SAR of the substituents on the aromatic ring groups was broadly similar to that observed in our earlier series.^8^ Substitution of one of the aromatic rings with a 3- or 4-halo substituent provided compounds with single digit nanomolar potency and high intrinsic activity (agonist efficacy relative to 1 μM iloprost as a positive control) in the IP cAMP assay and in some cases >100-fold selectivity over DP1 in the same assay platform. A 3- or 4-methoxy group (**5e** and **5j**) on one of the phenyl rings again provided analogues with single or double digit nanomolar potency and reasonable selectivity and was the most polar substituent that was tolerated. The in vitro potency and intrinsic activity of these monosubstituted analogues were comparable to data for **2** in our hands (Table 1) with a marginal improvement in functional selectivity with respect to DP1. An additional halogen substituent on either the same ring or the second aromatic ring did not improve receptor potency significantly and for a number of examples (**5q**–**u**) appeared to decrease efficacy. We were speculating at this stage that greater receptor efficacy may eventually offer an advantage for our series in further pharmacological measurements or in clinical testing, and so only compounds where there was a statistically significant improvement in intrinsic activity over **2** were considered for further studies.

### Early Compound Profiling

In addition to its function as a direct vasodilator, prostacyclin can also inhibit ADP-induced platelet aggregation. Such aggregated and activated platelets produce vasoactive substances such as thromboxane and serotonin that may cause harmful vasoconstriction and vascular remodeling.^13^ We thus measured the activity of our compounds to confirm the data from the recombinant receptor assay by testing in a primary human platelet aggregation assay as a potentially disease-relevant, physiological readout. This secondary assay was carried out as previously described,^14^ with aggregation induced by 2.5 μM ADP in the presence or absence of IP receptor agonists. The activity of all compounds in this assay was broadly reflective of their agonist effect in the cloned IP receptor assay with an approximate 2- to 10-fold difference in the observed potency. Again the potency of our compounds compared favorably to the inhibition IC_50_ observed for **2** (Table 1), although all compounds, regardless of efficacy in the cloned IP receptor assay, were able to achieve 100% inhibition of the aggregation response if tested at a high enough concentration.

We did note, however, that all of the compounds in our series had a rather poor effect when tested against the rat IP receptor, with EC_50_ values typically 10- to 30-fold weaker than for the human receptor, and we thus had some concerns about being able to demonstrate in vivo pharmacological activity in the rat. Despite this, several compounds with good human receptor potency and selectivity, which in general all had excellent stability in microsomes from both human and rat (Table 1), were surveyed for their PK properties in rat (Table 2).

**Table 2:** PK Parameters from Rat PK Screening of Selected IP Agonists^a^

compd	dose iv/po (mg/kg)	*C*_max_ (μg/mL)	*T*_max_ (h)	*T*_1/2_ (h)	*F* (%)	Cl_obs_ (L h^–1^ kg^–1^)	*V*_ss_ (L)	AUC_0–inf_(hr·μg/kg)
**5c**	2/10	3.7	1.5	6.7	57	0.43	2.71	14.6
**5e**	2/10	0.95	0.3	0.3	100	3.4	0.71	3.4
**5g**	2/10	3.4	0.3	3.4	39	1.2	2.74	7.9
**5j**	2/10	0.9	0.3	3.3	86	5.1	6.3	1.9
**5l**	2/10	1.2	0.3	3.9	100	1.8	6.2	7.9
**5m**	2.1/10.5	3.0	0.5	5	88	0.8	3.5	2.6
**5o**	2/10	1.43	0.833	3.2	24	0.6	1.1	4.5

a *C*_max_ = maximal plasma concentration reached after oral administration. *T*_max_ = time to reach the maximal plasma concentration after oral administration. *T*_1/2_ = terminal half-life after iv administration at 2 mg/kg. *F* = % oral bioavailability. AUC_0–inf_ = exposure (area under the curve) after oral administration. Cl_obs_ = total clearance rate. *V*_ss_ = volume of distribution.

On the basis of the combination of the in vitro activity/selectivity and the PK data obtained from this selection of compounds in particular the high *C*_max_ values, **5c**, **5g**, and **5m** were identified as three potential candidates for testing in our rat in vivo monocrotaline (MCT) model. The two methoxy substituted compounds (**5e** and **5j**) were discarded when we observed high clearance and a moderate *C*_max_ after oral administration in vivo. As the in vivo assay required chronic administration of compounds and was time and resource intensive, we were limited to the selection of two compounds to progress at this stage. Of the three compounds of interest remaining, **5c** had the highest total exposure (AUC), as well as longest half-life in the rat, which we felt provided the best opportunity to observe in vivo pharmacological activity. For the second selection we preferred **5g** over **5m**. Although both were more potent than **5c** at the rat receptor, which we thought may also be important, and each had excellent in vitro selectivity for the human IP receptor over the DP1 receptor, **5g** had significantly higher total exposure (AUC) in the rat PK study.

### Scale-Up and in Vivo Testing of Selected Compounds

**Scheme 2:**
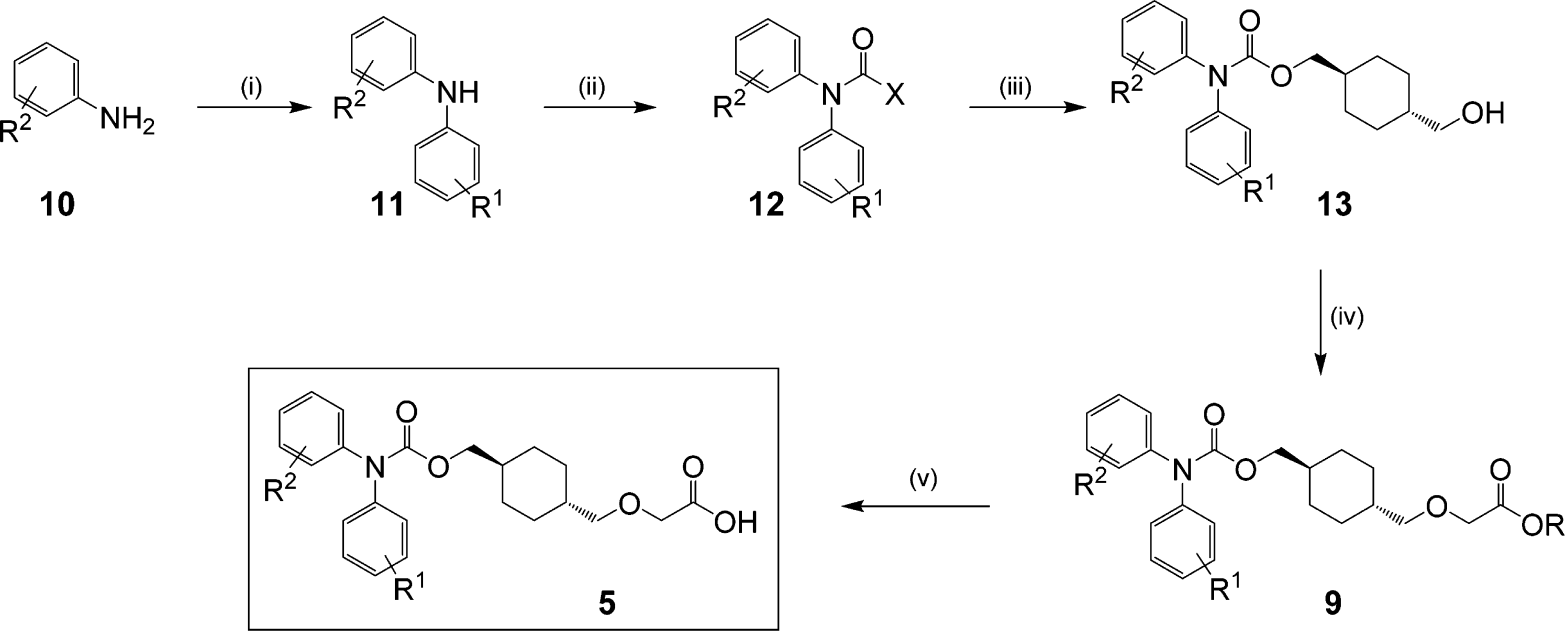
Larger Scale Syntheses of the Cyclohexyl Scaffold/Carbamate Series Reagents and conditions. Method 1: [R^1^ = 4-Cl or 3-F, R^2^ = H] (i) [R^2^ = 3-F] PhBr, KO*^t^*Bu, Pd(dppf)Cl_2_/CH_2_Cl_2_, toluene; (ii) triphosgene, pyridine, 0 °C; (iii) [X = Cl] (1*r*,4*r*)-cyclohexane-1,4-diyldimethanol, pyridine, heat; (iv) *tert*-butyl diazoacetate, (Rh(OAc)_2_)_2_ (v) HCl, dioxane. Method 2: [R^1^ = 4-Cl, R^2^ = H] (ii) carbonyldiimidazole, acetonitrile, K_3_PO_4_; (iii) [X = 1-imidazole] (1*r*,4*r*)-cyclohexane-1,4-diyldimethanol (4 equiv), acetonitrile, 65 °C (68–78% two steps); (iv) ethyl bromoacetate, tetrabutylammonium bromide, 50% NaOH, toluene; (v) (a) heat; (b) HCl (62–67% two steps).

For testing in the 21-day in vivo MCT model, the two compounds selected needed to be resynthesized on a multigram scale. Having already narrowed down the range of biaryl carbamate groups of interest to just two and in view of the unpredictable yields from the copper catalyzed arylation of **8**, we elected to start from the requisite biarylamine **10** (Scheme 2, method 1). For **5c**, the starting material 4-chloro-*N*-phenylaniline was commercially available and **11** (R^1^ = H, R^2^ = 3-F), the starting material for the preparation of **5g** which was not then readily available, was prepared by Buchwald coupling of 3-fluoroaniline (**10**, R^2^ = 3-F) with bromobenzene. Treatment of the biarylamines with triphosgene at 0 °C in pyridine provided the intermediates **12** (R^1^ = H, R^2^ = 4-Cl) and **12** (R^1^ = H, R^2^ = 3-F) that could be isolated. For the 3-fluoro analogue, the yield in the next reaction was enhanced by activation of this intermediate with DMAP, but in the case of **12** (R^1^ = H, R^2^ = 4-Cl) the chlorocarbamate was reacted directly with *trans*-1,4-cyclohexanedimethanol to provide **13** (R^1^ = H, R^2^ = 4-Cl). The sequence was completed with the carbene insertion reaction using *tert*-butyl diazoacetate followed by ester hydrolysis with HCl/dioxane.

**Figure 3:**
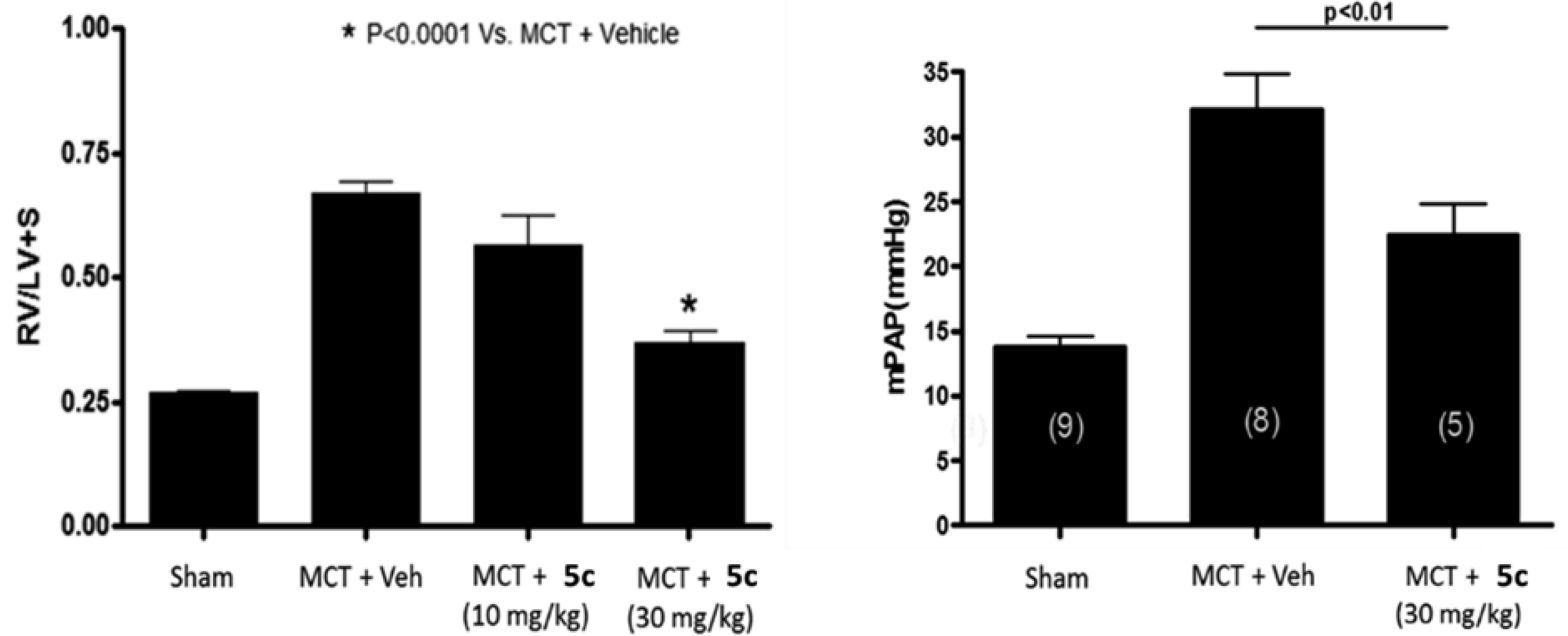
Effect of **5c** in the MCT model after oral administration. MCT = monocrotaline; Veh = vehicle; RV/(LV + S) = ratio of right ventricle weight to weight of left ventricle plus septum, an index of right ventricular hypertrophy (*n* = 8 for sham, *n* = 8 for MCT + veh group and *n* = 10 for **5c** treatment groups); mPAP = mean pulmonary arterial pressure.

When dosed in the MCT model in a preventative mode, both compounds demonstrated a dose-dependent inhibition of the increase in the ratio of right ventricular weight to the combined weight of left ventricle and septum (RV/(LV +S)) induced by administration of monocrotaline (data for **5c** are shown in Figure 3; data for **5g** are included in the Supporting Information), indicative of an inhibitory effect on the development of right ventricular hypertrophy. At the highest dose of 30 mg/kg twice daily for 21 consecutive days after the initiation of the insult with monocrotaline, RV/(LV + S) values were similar to those in sham controls. Supporting the observed effect on hypertrophy, **5c** was able to significantly reduce the MCT-induced increase in pulmonary arterial pressure in a subset of the test group (Figure 3) and pulmonary vessel wall thickness in 5 animals (see Supporting Information) at the highest dose of 30 mg/kg.

As might be expected from such highly lipophilic carboxylic acid compounds (clogP for **5c** = 5.6; clogP for **5g** = 5.0), plasma protein binding was very high (∼99%, i.e., 1% free fraction) for each across all species. The unbound *C*_max_ plasma concentrations for **5c** and **5g** after a single oral dose at 30 mg/kg were estimated to be 300 nM (based on a 1% free fraction of the measured *C*_max_ of a 30 mg/kg dose in a separate experiment designed to measure dose proportionality). Although agonists of some GPCRs with very efficient coupling can achieve full activation of the receptor at very low receptor occupancy, it is worth noting in this case that the unbound plasma concentrations in vivo were in the same range as the in vitro EC_50_ values for the two compounds at the recombinant rat IP receptor cAMP assay. In addition, the unbound *C*_max_ for **5c** following the ineffective 10 mg/kg dose was around 100 nM (as calculated from the PK experiment) which is 5-fold lower than the EC_50_ at the rat IP receptor. This provided a possible explanation for the lack of effect at this dose and allowed for a hypothesis as to the required efficacious human exposure requirements. It remains unclear however that the same correlation can be made in human, as the cAMP assay may not be the most relevant readout.

### Further Profiling

Encouraged by these data, we carried out more extensive profiling of **5c** and **5g**. In radioligand binding assays of affinity for the IP receptor in multiple species, there was little to choose between the two compounds (Table 3). In addition, **5c** and **5g** showed good binding selectivity over almost all the related prostaglandin receptor subtypes tested, with the exception of EP3, and in a wider screen no additional off-target liabilities were observed.^15^ Again, we noted clear species differences for the IP receptor affinities and the difference between the human and rat receptor was similar to that observed in the IP cAMP functional assays. In HTRF cAMP functional assays for other family members, no significant agonist activity was observed at the other receptors tested (EP2, EP4), suggesting that any functional activity arising from the modest binding affinity to these prostanoid receptors would most likely be in the antagonist direction. In contrast to our earlier series, the prostaglandin receptor for which **5c** and **5g** had the most significant receptor binding affinity was human EP3 (the EP3v6 form of the receptor was used for the study) and an EP3/IP binding selectivity ratio of 30- to 50-fold was observed. This was a surprise to us, as no previous series of IP agonists we had tested (including **2**) had any affinity for this receptor. Unlike IP, EP2, and EP4, EP3 is a G_i_ coupled receptor, and as a result functional selectivity is not directly comparable in a cAMP assay as forskolin activation is required making a functional selectivity comparison difficult to quantify. In our melanophore platform, however,^16^ both compounds behaved as EP3 agonists with potencies similar to their binding affinities (see Supporting Information data). Again, a true functional selectivity is difficult to deduce, but the data were similar to the agonist effect seen for iloprost in the EP3 melanophore assay. We were satisfied though, based on the binding (K_i_) data, that the receptor selectivity for these two compounds was adequate for us to proceed but with a requirement to monitor potential EP3 mediated effects.

**Table 3:** Radioligand Binding Assay Profiling of Potential Lead Compounds on Cloned Human Receptors and Prostacyclin Receptors from Other Preclinical Species

	*K*_i_ (μM)^a^		
binding assay (ligand)	**5c**	**5g**	iloprost
IP ([^3^H]-iloprost)	0.003 (*n* = 6; log SD = 0.34)	0.007 (*n* = 5; log SD = 0.28)	0.0032 (*n* = 25; log SD = 0.4)
DP_1_ ([^3^H]-PGD2)	2.6	1.85	nd
EP_1_ ([^3^H]-PGE2)	9.6	nd	nd
EP_2_ ([^3^H]-PGE2)	0.610	0.57	nd
EP_3v6_ ([^3^H]-PGE2)	0.143 (*n* = 3; log SD = 0.07)	0.210 (*n* = 3; log SD = 0.3)	3.7
EP_4_ ([^3^H]-PGE2)	0.678	1.2	nd
rat IP ([^3^H]-iloprost)	0.076 (*n* = 3; log SD = 0.21)	0.051 (*n* = 3; log SD = 0.2)	0.001 (*n* = 3; log SD = 0.09)
dog IP ([^3^H]-iloprost)	0.256 (*n* = 5; log SD = 0.16)	0.183 (*n* = 3; log SD = 0.23)	0.0017 (*n* = 8; log SD = 0.13)
monkey IP ([^3^H]-iloprost)	0.0012 (*n* = 6; log SD = 0.16)	0.0026 (*n* = 3; log SD = 0.07)	0.0016 (*n* = 11; log SD = 0.13)

a Data are the mean of two determinations unless otherwise stated.

Since GPCR functional assays performed in cells expressing high levels of recombinant receptors can often be influenced by receptor reserve effects, which may exaggerate potency and efficacy, further studies at the human IP receptor were performed to carefully characterize the in vitro potency and efficacy of **5c** and **5g**. Thus, a series of assays were performed in which receptor expression levels were systematically reduced. In addition, cAMP assays were performed in primary human pulmonary arterial smooth muscle cells. When we reached a level of no receptor reserve in the recombinant assay, the potency and efficacy data were essentially identical in each system (Supporting Information). In both of these assay systems, as well as the screening assay, **5c** showed significantly greater potency and efficacy than **2**. Further assays in pulmonary arterial smooth muscle cells (PASMC) from PAH patients have recently been reported that also showed a greater efficacy for **5c** in increasing cAMP levels compared to **2** (an effect that was blocked for both compounds by the IP antagonist RO-1138452), although there was no difference in EC_50_.^17^

Finally, as part of our in vitro evaluations, neither **5c** nor **5g** showed any measurable inhibition of the most highly expressed cytochrome P450 enzymes (IC_50_ > 50 μM for CYPs 1A2, 2D6, 3A4 2C8, 2C9, and 2C19) and no inhibition of hERG channel functional activity was observed in a patch clamp assay (IC_50_ > 30 μM).

### Selection of a Development Candidate

The first meaningful differentiation between the two compounds was observed during our early solid state characterization. Surprisingly, although the structures of the two compounds were very similar, both the free acid (mp ∼128 °C) and the sodium salt (mp ∼243 °C) of **5c** were crystalline and nonhygroscopic solids, whereas the free acid of **5g** was an oil and the sodium salt was a low melting monohydrate (see Supporting Information). It was on the basis of these data that the 4-chloro analogue **5c** was selected for further investigation as it had two potential solid state forms that met our in-house solid state screening developability criteria. **5g** on the other hand had only a single acceptable solid form which, being a hydrate, may have had a higher likelihood of running into issues in development. In addition, when we investigated further scale up synthesis methods, all key synthetic intermediates for **5c** could be isolated as solids. This was not the case for **5g** which might also have presented significant development challenges.

**5c** was subsequently prepared in kilogram amounts using some modifications to the first scale-up route, primarily directed toward improving early process safety by the replacement of phosgene and the carbene insertion reactions with more benign alternatives (Scheme 2, method 2) and toward shortening the overall procedure by telescoping some steps. Hence, **11** (R^1^ = H, R^2^ = 4-Cl) was treated with 1,1′-carbonyldiimidazole (CDI) in acetonitrile in the presence of potassium phosphate. After this first reaction was complete, the intermediate **12** (R^1^ = H, R^2^ = 4-Cl) was not isolated but rather excess 1,4-*trans*-cyclohexane dimethanol was added to the reaction mixture to provide **13** (R^1^ = H, R^2^ = 4-Cl) which was isolated by removal of acetonitrile and precipitation from water. **13** (R^1^ = H, R^2^ = 4-Cl) was alkylated with *tert*-butyl bromoacetate utilizing 50% aqueous sodium hydroxide and tetrabutylammonium bromide in toluene. On reaction completion, the intermediate *tert*-butyl ester was simply heated to hydrolyze the ester. The resultant acid was isolated as the sodium salt which crystallized from aqueous acetone. The sodium salt could then be acidified with hydrochloric acid and the free acid of **5c** isolated by filtration with purity in excess of 99%.^18^

With now a significant amount of material in hand we were able to collect additional data to confirm the nomination of **5c** for further development. In PK studies across a number of preclinical species **5c** exhibited dose-dependent exposure with good bioavailability after oral administration in all species tested (Table 4 and Supporting Information). Particularly noteworthy was the concentration–time profile and long elimination half-life in the monkey. **5c** appeared to have a very low peak-to-trough ratio in the range of 3–5 following oral administration, suggesting that this compound might have an advantageous profile in the clinic. As the majority of the side effects observed with prostacyclin therapies, such as flushing, systemic hypotension, and jaw pain, are believed to be largely on target, they may be greatly ameliorated by such minimal fluctuations in plasma exposure if these can be confirmed in human studies. Such tight control of plasma levels may therefore be expected to provide improved tolerability compared to existing prostacyclin treatments. In addition, the long half-life in preclinical species also strongly suggested the possibility for once daily oral formulation.

**Table 4:** Pharmacokinetic Parameters of **5c** after Administration to Male CD-1 Mice, Sprague-Dawley Rats, Beagle Dogs, and Cynomolgus Monkeys^a^

(1) After Intravenous Administration					
species	iv dose (mg/kg)	*T*_1/2_ (h)	Cl_obs_ (L h^–1^ kg^–1^)	*V*_ss_ (L)	AUC_0–inf_ (h·μg/mL)
mouse	1.9	5.59	0.689	3.66	2.76
rat	2	6.72 ± 0.98	0.427 ± 0.163	2.74 ± 1.11	5.09 ± 1.62
dog	0.2	3.54 ± 0.42	0.202 ± 0.057	0.308 ± 0.050	1.05 ± 0.30
monkey	0.1	24.2 ± 2.1	0.881 ± 0.579	10.1 ± 0.6	0.145 ± 0.095

(2) After Oral Administration						
species	po dose (mg/kg)	*T*_1/2_ (h)	*C*_max_ (μg/mL)	*T*_max_ (h)	AUC_0–inf_ (h·μg/mL)	*F* (%)
mouse	9.49	8.08	4.94	0.250	11.5	83.4
rat	10.0	5.45 ± 1.52	3.70 ± 1.34	1.50 ± 0.87	14.6 ± 3.41	57.4
dog	0.2	9.79 ± 0.94	0.327 ± 0.016	0.417 ± 0.144	0.719 ± 0.290	68.4
	2	5.53 ± 1.48	3.44 ± 0.90	0.833 ± 0.289	9.75 ± 5.27	92.9
monkey	0.1	17.5 ± 3.4	0.0121 ± 0.0036	2.67 ± 2.89	0.161 ± 0.116	98.7
	0.5	38.7 ± 21.3	0.0928 ± 0.0297	4.17 ± 3.18	2.90 ± 1.35	>100

a *C*_max_ = maximal plasma concentration reached after oral administration. *T*_max_ = time to reach the maximal plasma concentration after oral administration. *T*_1/2_ = terminal half-life after iv administration at 2 mg/kg. *F* = % oral bioavailability. AUC_0–inf_ = exposure (area under the curve). Cl_obs_ = total clearance rate. *V*_ss_ = volume of distribution.

One explanation for the prolonged exposure was the possibility of enterohepatic recirculation. This suggestion was inferred from concentration–time profiles in PK studies across species in which a second, delayed absorption phase was routinely observed. A more definitive study was therefore conducted in bile-duct cannulated (BDC) rats. After an intravenous administration of **5c** to either normal or BDC rats, the second absorption phase was eliminated in the BDC rats with an accompanying 3-fold reduction in the apparent plasma half-life, supporting the suggestion of recycling of **5c** back to the plasma via bile excretion into the gut in normal rats. Concentrations of **5c** were negligible in bile and urine compared to plasma indicating that the major path of elimination was hepatic metabolism and that a conjugated species may be involved.

**Scheme 3:**
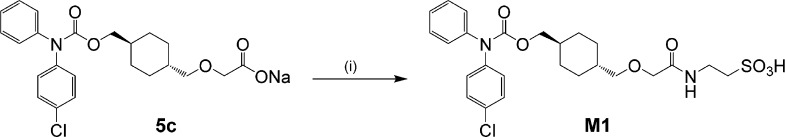
Synthetic Preparation of M1 Metabolite Reagents and conditions: (i) (a) thionyl chloride; (b) taurine, 20% NaOH, 0 °C.

**Figure 4:**
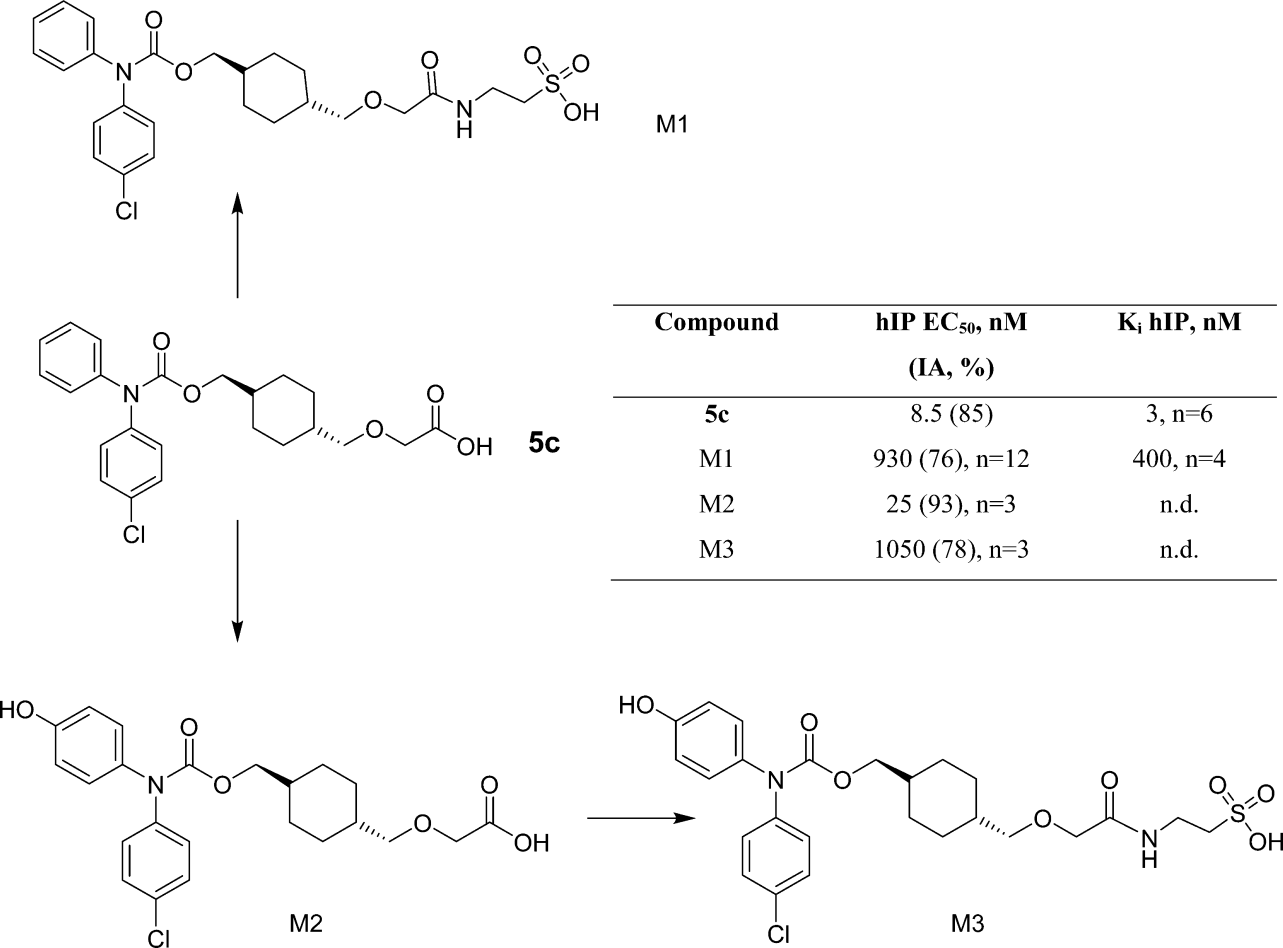
Metabolite profile of **5c** in the rat.

A taurine-conjugated metabolite was subsequently identified in bile (M1, Figure 4), which was also observed in circulating plasma, although at much lower concentrations (∼5%) compared to parent. Further, two oxidized products, one of which was also a taurine-conjugate, were identified (M2 and M3) in plasma. Both conjugated metabolites of **5c** had weak activity at the IP receptor (Figure 4), whereas the low level metabolite M2 unexpectedly had double-digit nanomolar functional activity at the human receptor. It is known that taurine can be conjugated to bile acids and these conjugates may be hydrolyzed in the gut which allows for bile acid reabsorbtion.^19^ Having observed a taurine conjugate metabolite, we hypothesized that **5c**, which like bile acids is also a highly lipophilic carboxylic acid, could also be a substrate for the same recirculation mechanism. To confirm that the **5c** taurine conjugate was a potential source of the observed recirculation, we synthesized M1 (Scheme 3). When dosed orally to rats essentially identical plasma levels of **5c** were obtained compared to dosing of the parent compound. To eliminate the potential for a simple acidic hydrolysis of the conjugate in the GI tract to produce **5c** which could be absorbed directly, we incubated M1 in simulated gastric fluid at room temperature for 3 h with little chemical degradation to the parent. It is therefore highly likely that this conjugation and recirculation process contributes significantly to the favorable pharmacokinetic profile. Interestingly, no analogous conjugate was observed in vivo following dosing of **5g**, further highlighting the differences in behavior of the two compounds despite their very similar structures.

## Conclusions

**5c** is an orally bioavailable, non-prostanoid IP receptor agonist that is efficacious in the rat MCT model of PAH. It has good selectivity in both binding and functional assays with respect to most members of the prostanoid receptor family, but a more modest 30-50-fold selectivity over the EP3 receptor. In our hands, its potency and efficacy are superior in vitro to **2** (the active metabolite of the clinical compound **1**) in in vitro measures of IP receptor mediated cAMP signaling and it is more potent in inhibiting ADP-induced aggregation in ex vivo platelet assays. **5c** is rapidly absorbed after oral administration in preclinical species, and its systemic clearance is primarily due to hepatic metabolism with probable enterohepatic recirculation. The elimination half-life ranges from 5 to 8 h in rodents to 10 and 18 h in dog and monkey, respectively. Exposure was so prolonged in the monkey that it may provide very low peak to trough plasma concentration ratios with repeated dosing in patients that could closely mimic continuous intravenous infusion. On the basis of these data, as well as an acceptable safety profile in IND-enabling studies, **5c** as its free acid solid form (which was subsequently given the USAN name ralinepag) was selected for further development for the treatment of PAH. Clinical studies are underway to determine if the excellent in vitro and PK profile can provide improvements in clinical efficacy over current therapies.

## Experimental Section

### Chemistry

Proton nuclear magnetic resonance (^1^H NMR) spectra were recorded on a Bruker Avance-400 equipped with a QNP (Quad Nucleus Probe) or a BBI (Broad Band Inverse) and *z*-gradient. Chemical shifts are given in parts per million (ppm) with the residual solvent signal used as reference. NMR abbreviations are used as follows: s = singlet, d = doublet, dd = doublet of doublet, ddd = doublet of doublet of doublets, dt = doublet of triplet, t = triplet, tt = triplet of triplets, q = quartet, m = multiplet, br = broad. Microwave irradiations were carried out using either an Initiator or Initiator+ machine (Biotage). Thin-layer chromatography (TLC) was performed on silica gel 60 F_254_ (Merck), preparatory thin-layer chromatography (prep TLC) was preformed on PK6F silica gel 60 A 1 mm plates (Whatman), and column chromatography was carried out either manually on a silica gel column using Kieselgel 60, 0.063–0.200 mm (Merck), or using prepacked columns for Isolera 1 (Biotage). Evaporation was done in vacuo on a Buchi rotary evaporator.

Test compound purity was measured by UV (254 nm) peak area over a 5 min 0–100% acetonitrile gradient elution. LCMS specs were the following. (1) PC: HPLC-pumps, LC-10AD VP, Shimadzu Inc.; HPLC system controller, SCL-10A VP, Shimadzu Inc.; UV detector, SPD-10A VP, Shimadzu Inc.; autosampler, CTC HTS, PAL, Leap Scientific; mass spectrometer, API 150EX with Turbo Ion Spray source, AB/MDS Sciex; software, Analyst 1.2. (2) Mac: HPLC-pumps, LC-8A VP, Shimadzu Inc.; HPLC system controller, SCL-10A VP; Shimadzu Inc.; UV detector, SPD-10A VP, Shimadzu Inc.; autosampler, 215 Liquid Handler, Gilson Inc.; mass spectrometer, API 150EX with Turbo Ion Spray source; AB/MDS Sciex Software, Masschrom 1.5.2. All test compounds were of greater than 95% purity by one or more of the above methods, and LC–MS purity for individual compounds is noted in the experimental.

### Small Scale Synthesis Methods

Methods for the preparation of *trans* isomers are described. Identical methods were used for the *cis* isomers where prepared using (1*s*,4*s*)-cyclohexane-1,4-diyldimethanol in place of (1*r*,4*r*)-cyclohexane-1,4-diyldimethanol.

#### Preparation of 2-(((1*r*,4*r*)-4-((Diphenylcarbamoyloxy)methyl)cyclohexyl)methoxy)acetic Acid (**5a**)

The title compound was obtained from *tert*-butyl 2-(((1*r*,4*r*)-4-((phenylcarbamoyloxy)methyl)cyclohexyl)methoxy)acetate and iodobenzene, using a similar method to the one described for compound **5e**. LCMS *m*/*z* = 398.10 [M + H]^+^; purity, 98%; ^1^H NMR (400 MHz, DMSO-*d*_6_) δ 1.10–1.42 (m, 8H), 1.53–1.60 (m, 2H), 1.70–1.87 (m, 2H), 3.91–3.93 (d, *J* = 4.2 Hz, 2H), 3.98 (s, 2H), 7.20–7.31 (m, 6H), 7.30–7.50 (m, 4H).

#### Preparation of 2-(((1*s*,4*s*)-4-((Diphenylcarbamoyloxy)methyl)cyclohexyl)methoxy)acetic Acid (**5b**)

The title compound was obtained from *tert*-butyl 2-(((1*s*,4*s*)-4-((phenylcarbamoyloxy)methyl)cyclohexyl)methoxy)acetate and iodobenzene, using a similar method to the one described for compound **5e**. LCMS *m*/*z* = 398.45 [M + H]^+^; purity, 99%; ^1^H NMR (400 MHz, DMSO-*d*_6_) δ 1.15–1.40 (m, 8H), 1.50–1.62 (m, 2H), 1.75–1.81 (m, 2H), 3.90–3.92 (d, *J* = 4.3 Hz, 2H), 3.96 (s, 2H), 7.21–7.32 (m, 6H), 7.35–7.39 (m, 4H).

#### Preparation of 2-(((1*s*,4*s*)-4-(((4-Chlorophenyl)(phenyl)carbamoyloxy)methyl)cyclohexyl)methoxy)acetic Acid (**5d**)

The title compound was obtained from *tert*-butyl 2-(((1*s*,4*s*)-4-((phenylcarbamoyloxy)methyl)cyclohexyl)methoxy)acetate and 1-chloro-4-iodobenzene, using a similar method to the one described for compound **5e**. LCMS *m*/*z* = 432.1 [M + H]^+^; purity, 99%; ^1^H NMR (400 MHz, DMSO-*d*_6_) δ ppm 1.28–1.53 (m, 8H), 1.60–1.81 (m, 2H), 3.29 (d, *J* = 7.07 Hz, 2H), 3.96 (s, 2H), 3.98 (d, *J* = 6.69 Hz, 2H), 7.24–7.33 (m, 5H), 7.36–7.48 (m, 4H).

#### Preparation of 2-(((1*r*,4*r*)-4-(((4-Methoxyphenyl)(phenyl)carbamoyloxy)methyl)cyclohexyl)methoxy)acetic Acid (**5e**). Step A: Preparation of ((1*r*,4*r*)-4-(Hydroxymethyl)cyclohexyl)methyl Phenylcarbamate

To a solution of (1*r*,4*r*)-cyclohexane-1,4-diyldimethanol (5 g, 34.7 mmol) in pyridine at room temperature was added phenyl isocyanate (4.13 g, 34.7 mmol). The reaction mixture was stirred for 5 h, concentrated, and extracted with ethyl acetate. The extract was dried over MgSO_4_ and concentrated in vacuo. The residue was purified by silica gel column chromatography to give the title compound (4.69 g, 51.5%). LCMS *m*/*z* = 264.43 [M + H]^+^; ^1^H NMR (400 MHz, DMSO-*d*_6_) δ ppm 0.81- 1.09 (m, 4H), 1.30–1.39 (m, 1H), 1.51–1.62 (m, 1H), 1.75–1.88 (m, 4H), 3.15–3.25 (d, *J* = 5.8 Hz, 2H), 3.82–3.95 (d, *J* = 6.56 Hz, 2H), 4.52 (t, *J* = 5.31 Hz, 1H), 6.29 (m, 1H), 7.30 (m, 2H), 7.48 (m, 2H), 9.62 (s, 1H).

#### Step B: Preparation of *tert*-Butyl 2-(((1*r*,4*r*)-4-((Phenylcarbamoyloxy)methyl)cyclohexyl)methoxy)acetate

To a solution of ((1*r*,4*r*)-4-(hydroxymethyl)cyclohexyl)methyl phenylcarbamate (2.5 g, 9.49 mmol) and diacetoxyrhodium (0.210 g, 0.475 mmol) in dichloromethane (50 mL) at 0°C was added dropwise a solution of *tert*-butyl 2-diazoacetate (1.350 g, 9.49 mmol) in dichloromethane (5 mL) over 20 min. After stirring for 30 min at room temperature, the solid was removed by filtration and the filtrate concentrated under reduced pressure. The residue was purified by silica gel column chromatography to give the title compound (3.32 g, 93%). LCMS *m*/*z* = 378.43 [M + H]^+^; ^1^H NMR (400 MHz, DMSO-*d*_6_) δ ppm 0.85–1.08(m, 4H), 1.42 (s, 9H), 1.41–1.62 (m, 2H), 1.78–1.81 (m, 4H), 3.25 (d, *J* = 6.3 Hz, 2H), 3.92 (d, *J* = 4.6 Hz, 2H), 6.29 (m, 1H), 7.31 (m, 2H), 7.48 (m, 2H), 9.62 (s, 1H).

#### Step C: Preparation of 2-(((1*r*,4*r*)-4-(((4-Methoxyphenyl)(phenyl)carbamoyloxy)methyl)cyclohexyl)methoxy)acetic Acid

*tert*-Butyl 2-(((1*r*,4*r*)-4-((phenylcarbamoyloxy)methyl)cyclohexyl)methoxy)acetate: (50.0 mg, 0.132 mmol), copper(I) iodide (12.61 mg, 0.066 mmol), K_3_PO_4_ (56.2 mg, 0.265 mmol), 4-methoxyphenyl iodide (31.0 mg, 0.132 mmol), and dioxane (1.6 mL) were added to a microwave vial. The reaction mixture was heated under microwave irradiation at 150 °C for 5 h. The cooled mixture was filtered through a plug of MgSO_4_. The solvent was evaporated, and the resulting oil was redissolved in HCl (4 M in dioxane, 1.987 mmol), and the mixture stirred overnight at room temperature After removal of the solvent, the residue was purified by preparative LCMS to provide the title compound as a white solid (12.2 mg, 21.6%). LCMS *m*/*z* = 428.4 [M + H]^+^, purity, 99%; ^1^H NMR (400 MHz, CD_3_OD) δ ppm 0.92–0.97(m, 4H), 1.22–1.25 (m, 2H), 1.47–1.79 (m, 4H), 3.15 (d, *J* = 6.5 Hz, 2H), 3.79 (s, 3H), 3.95 (d, *J* = 6.0 Hz, 2H), 4.02 (s, 2H), 6.90–7.33 (m, 9H).

#### Preparation of 2-(((1*s*,4*s*)-4-(((4-Methoxyphenyl)(phenyl)carbamoyloxy)methyl)cyclohexyl)methoxy)acetic Acid (**5f**)

The title compound was obtained from *tert*-butyl 2-(((1*s*,4*s*)-4-((phenylcarbamoyloxy)methyl)cyclohexyl)methoxy)acetate and 1-iodo-4-methoxybenzene, using a similar method to the one described for compound **5e**. LCMS *m*/*z* = 428.2 [M + H]^+^; purity, 99%; ^1^H NMR(400 MHz, DMSO-*d*_6_) δ ppm 1.34–1.56 (m, 8H), 1.70–1.88 (m, 2H), 3.31 (s, 3H), 3.39 (d, *J* = 7.07 Hz, 2H), 4.00 (d, *J* = 7.20 Hz, 2H), 4.11 (s, 2H), 6.94–7.01 (m, 2H), 7.23–7.31 (m, 4H), 7.42–7.49 (m, 3H).

#### Preparation of 2-(((1*s*,4*s*)-4-(((3-Fluorophenyl)(phenyl)carbamoyloxy)methyl)cyclohexyl)methoxy)acetic Acid (**5h**)

The title compound was obtained from *tert*-butyl 2-(((1*s*,4*s*)-4-((phenylcarbamoyloxy)methyl)cyclohexyl)methoxy)acetate and 1-fluoro-3-iodobenzene, using a similar method to the one described for compound **5e**. LCMS *m*/*z* = 416.4 [M + H]^+^; purity, 99%; ^1^H NMR(400 MHz, DMSO-*d*_6_) δ ppm 1.28- 1.42 (m, 8H), 1.71 (s, 2H), 3.28 (d, *J* = 7.07 Hz, 2H), 3.96 (s, 2H), 3.99 (d, *J* = 6.44 Hz, 2H), 7.03- 7.11 (m, 2H), 7.20–7.33 (m, 4H), 7.37–7.43 (m, 3H).

#### Preparation of 2-(((1*r*,4*r*)-4-(((3-Chlorophenyl)(phenyl)carbamoyloxy)methyl)cyclohexyl)methoxy)acetic Acid (**5i**)

The title compound was obtained from *tert*-butyl-2-(((1*r*,4*r*)-4-((phenylcarbamoyloxy)methyl)cyclohexyl)methoxy)acetate and 1-chloro-3-iodobenzene, using a similar method to the one described for compound **5e**. LCMS *m*/*z* = 432.6 [M + H]^+^; purity, 99%.

#### Preparation of 2-(((1*r*,4*r*)-4-(((3-Methoxyphenyl)(phenyl)carbamoyloxy)methyl)cyclohexyl)methoxy)acetic Acid (**5j**)

The title compound was obtained from *tert*-butyl 2-(((1*r*,4*r*)-4-((phenylcarbamoyloxy)methyl)cyclohexyl)methoxy)acetate and 1-bromo-3-methoxybenzene, using a similar method to the one described for compound **5e**. LCMS *m*/*z* = 428.3 [M + H]^+^; purity, 98%; 1H NMR (400 MHz, DMSO-*d*_6_) δ ppm 0.81–0.98(m, 4H), 1.35–1.55 (m, 2H), 1.60–1.75 (m, 4H), 3.15 (d, *J* = 6.5 Hz, 2H), 3.50 (S, 2H), 3.75 (S, 3H), 3.95 (d, *J* = 6.0 Hz, 2H), 6.80–6.95 (m, 4H), 7.25–7.45 (m, 5H).

#### Preparation of 2-(((1*r*,4*r*)-4-((Phenyl(*p*-tolyl)carbamoyloxy)methyl)cyclohexyl)methoxy)acetic Acid (**5k**)

The title compound was obtained as a white solid from *tert*-butyl 2-(((1*r*,4*r*)-4-((phenylcarbamoyloxy)methyl)cyclohexyl)methoxy acetate and 1-iodo-4-methylbenzene using a similar method to the one described for compound **5e**. LCMS *m*/*z* = 412.2 [M + H]^+^ purity, 99%.

#### Preparation of 2-(((1*r*,4*r*)-4-(((4-Fluorophenyl)(phenyl)carbamoyloxy)methyl)cyclohexyl)methoxy)acetic Acid (**5l**)

The title compound was obtained as a white solid from *tert*-butyl-2-(((1*r*,4*r*)-4-((phenylcarbamoyloxy)methyl)cyclohexyl)methoxy)acetate and 1-fluoro-4-iodobenzene, using a similar method to the one described for compound **5e**. LCMS *m*/*z* = 416.5 [M + H]^+^; purity, 99%.; ^1^H NMR (400 MHz, methanol-*d*_4_) δ ppm 0.79–1.08 (m, 4H), 1.45–1.60 (m, 2H), 1.61–1.73 (m, 2H), 1.77–1.89 (m, 2H), 3.29 (d, *J* = 6.57 Hz, 2H), 15 3.83 (s, 2H), 3.97 (d, *J* = 6.06 Hz, 2H), 7.07–7.14 (m, 2H), 7.21–7.34 (m, 5H), 7.35–7.41 (m, 2H).

#### Preparation of 2-(((1*r*,4*r*)-4-(((4-Chloro-3-fluorophenyl)(phenyl)carbamoyloxy)methyl)cyclohexyl)methoxy)acetic Acid (**5m**)

The title compound was obtained as a white solid from *tert*-butyl 2-(((1*r*,4*r*)-4-((phenylcarbamoyloxy)methyl)cyclohexyl)methoxy)acetate and 1-chloro-2-fluoro-4-iodobenzene, using a similar method to the one described in compound **5e**. LCMS *m*/*z* = 450.1 [M + H]^+^; purity, 99%; ^1^H NMR (400 MHz, methanol-*d*_4_) δ ppm 0.69–0.91 (m, 4H), 1.08–1.31 (m, 1H), 1.32–1.48 (m, 1H), 1.48–1.64 (m, 2H), 1.64–1.81 (m, 2H), 3.17 (d, *J* = 6.57 Hz, 2H), 3.71 (s, 2H), 3.87 (d, *J* = 6.06 Hz, 2H), 6.94 (ddd, *J* = 8.75, 2.43, 1.20 Hz, 1H), 7.14–7.24 (m, 4H), 7.27–7.34 (m, 3H).

#### Preparation of 2-(((1*r*,4*r*)-4-(((3-Chloro-4-fluorophenyl)(phenyl)carbamoyloxy)methyl)cyclohexyl)methoxy)acetic Acid (**5n**)

The title compound was obtained as a white solid from *tert*-butyl 2-(((1*r*,4*r*)-4-((phenylcarbamoyloxy)methyl)cyclohexyl)methoxy)acetate and 2-chloro-1-fluoro-4-iodobenzene, using a similar method to the one described in compound **5e**. LCMS *m*/*z* = 450.2 [M + H]^+^; purity, 99%.

#### Preparation of 2-(((1*r*,4*r*)-4-(((3,4-Difluorophenyl)(phenyl)carbamoyloxy)methyl)cyclohexyl)methoxy)acetic Acid (**5o**)

The title compound was obtained as a white solid from *tert*-butyl 2-(((1*r*,4*r*)-4-((phenylcarbamoyloxy)methyl)cyclohexyl)methoxy)acetate and 1,2-difluoro-4-iodobenzene, using a similar method to the one described in compound **5e**. LCMS *m*/*z* = 434.5 [M + H]^+^; purity, 99%; ^1^H NMR (400 MHz, methanol-*d*_4_) δ ppm 0.93–1.11 (m, 4H), 1.52–1.68 (m,2H), 1.67–1.81 (m, 2H), 1.84–1.98 (m, 2H), 3.36 (d, *J* = 6.44 Hz, 2H), 3.90 (s, 2H), 4.05 (d, *J* = 6.06 Hz, 2H), 7.10–7.18 (m, 1H), 7.27–7.42 (m, 5H), 7.45–7.53 (m, 2H).

#### Preparation of 2-(((1*r*,4*r*)-4-(((3-Fluorophenyl)(4-methoxyphenyl)carbamoyloxy)methyl)cyclohexyl)methoxy)acetic Acid (**5p**). Step A: Preparation of *tert*-Butyl 2-(((1*r*,4*r*)-4-(Hydroxymethyl)cyclohexyl)methoxy)acetate

To a solution of (1*r*,4*r*)-cyclohexane-1,4-diyldimethanol (5.0 g, 34.7 mmol) in benzene (20 mL) at room temperature were added tetrabutylammonium iodide (6.40 g, 17.34 mmol) and 50% aqueous NaOH (10 mL, 34.7 mmol). The mixture was stirred vigorously for 5 min, and then *tert*-butyl 2-bromoacetate (5.63 mL, 38.1 mmol) was added. The reaction was stirred vigorously for a further 2 h. The mixture was partitioned between 50% aqueous NaOH (100 mL) and EtOAc (100 mL). The aqueous layer was extracted again with EtOAc (100 mL). The combined organic layer was dried and concentrated. The residue was purified by silica gel column chromatography to provide the title compound as a colorless oil (3.96g, 44%). LCMS *m*/*z* = 259.3 [M + H]^+^; ^1^H NMR (400 MHz, CDCl_3_) δ ppm 0.89–1.06 (m, 4H), 1.47 (s, 9H), 1.55–1.68 (m, 2H), 1.76–1.98 (m, 4H), 3.32 (d, *J* = 6.57 Hz, 2H), 3.45 (d, *J* = 6.32 Hz, 2H), 3.93 (s, 2H).

#### Step B: Preparation of *tert*-Butyl 2-(((1*r*,4*r*)-4-((3-Fluorophenylcarbamoyloxy)methyl)cyclohexyl)methoxy)acetate

To a solution of *tert*-butyl 2-(((1*r*,4*r*)-4-(hydroxymethyl)cyclohexyl)methoxy)acetate (1.0 g, 3.87 mmol) and pyridine (0.438 mL, 5.42 mmol) in CH_2_Cl_2_ (10 mL) was added 3-fluorophenyl isocyanate (0.480 mL, 4.26 mmol), and the reaction was stirred at room temperature overnight. The reaction was then heated to reflux for 5 h. After removal of the solvent, the residue was purified by silica gel column chromatography to yield the title compound as a white solid (1.12 g). LCMS *m*/*z* = 340.4 [M – *tert*-butyl + H]^+^; ^1^H NMR (400 MHz, CDCl_3_) δ ppm 0.85–1.04 (m, 4H), 1.41 (s, 9H), 1.51–1.64 (m, 2H), 1.69–1.87 (m, 4H), 3.26 (d, *J* = 6.32 Hz, 2H), 3.87 (s, 2H), 3.92 (d, *J* = 6.57 Hz, 2H), 6.57 (s, 1H), 6.68 (dt, *J* = 8.34, 2.53 Hz, 1H), 6.94 (d, *J* = 8.59 Hz, 1H), 7.13–7.18 (m, 1H), 7.20–7.28 (m, 1H).

#### Step C: Preparation of 2-(((1*r*,4*r*)-4-(((3-Fluorophenyl)(4-methoxyphenyl)carbamoyloxy)methyl)cyclohexyl)methoxy)acetic Acid

The title compound was obtained as a white solid from 1-iodo-4-methoxybenzene and *tert*-butyl 2-(((1*r*,4*r*)-4-((3-fluorophenylcarbamoyloxy)methyl)cyclohexyl)methoxy)acetate, using a similar method to the one described for compound **5e**. LCMS *m*/*z* = 446.5 [M + H]^+.^; purity, 99%; ^1^H NMR (400 MHz, DMSO-*d*_6_) δ ppm 0.86 (t, *J* = 11 Hz, 4H), 1.39 (s, 2H), 1.54–1.62 (m, 2H), 1.64–1.74 (m, 2H), 3.23 (d, *J* = 6.32 Hz, 2H), 3.76 (s, 3H), 3.89 (d, *J* = 6.19 Hz, 2H), 3.94 (s, 2H), 6.91–6.98 (m, 2H), 6.99–7.07 (m, 2H), 7.17–7.26 (m, 3H), 7.36 (dt, *J* = 8.18, 6.88 Hz, 1H), 12.52 (br s, 1H).

#### Preparation of 2-(((1*r*,4*r*)-4-(((4-Chlorophenyl)(3-fluorophenyl)carbamoyloxy)methyl)cyclohexyl)methoxy)acetic Acid (**5q**)

The title compound was obtained as a white solid from 1-chloro-4-iodobenzene and *tert*-butyl 2-(((1*r*,4*r*)-4-((3-fluorophenylcarbamoyloxy)methyl)cyclohexyl)methoxy)acetate, using a similar method to the one described for compound **5e**. LCMS *m*/*z* = 450.5 [M + H]^+^. purity, 99%; ^1^H NMR (400 MHz, DMSO-*d*_6_) δ ppm 0.77–0.98 (m, 4H), 1.34–1.42 (m, 1H), 1.42–1.52 (m, 1H), 1.53–1.63 (m, 2H), 1.63–1.75 (m, 2H), 3.23 (d, *J* = 6.44 Hz, 2H), 3.91 (d, *J* = 6.06 Hz, 2H), 3.94 (s, 2H), 7.04–7.14 (m, 2H), 7.23–7.28 (m, 1H), 7.29–7.36 (m, 2H), 7.36–7.42 (m, 1H), 7.42–7.49 (m, 2H), 12.52 (br s, 1H).

#### Preparation of 2-(((1*r*,4*r*)-4-((Bis(4-fluorophenyl)carbamoyloxy)methyl)cyclohexyl)methoxy)acetate (**5r**). Step A: Preparation of Methyl 4-Fluoro-2-((1*r*,4*r*)-4-(hydroxymethyl)cyclohexyl)phenylcarbamate

4-Fluorophenyl isocyanate (4.75 g, 34.7 mmol), (1*r*,4*r*)-cyclohexane-1,4-diyldimethanol (5.0 g, 34.7 mmol), and pyridine (3.93 mL, 48.5 mmol) were dissolved in CH_2_Cl_2_ (30 mL). The reaction mixture was stirred at room temperature overnight. After removal of the solvent, the residue was purified by silica gel column chromatography to yield the title compound as a white solid (4.92 g, 50%). LCMS *m*/*z* = 282.4 [M + H]^+^; ^1^H NMR (400 MHz, DMSO-*d*_6_) δ ppm 0.81–1.07 (m, 4H), 1.25–1.38 (m, 1H), 1.49–1.64 (m, 1H), 1.72–1.82 (m, 4H), 3.19–3.24 (m, 2H), 3.89 (d, *J* = 6.57 Hz, 2H), 4.34 (t, *J* = 5.31 Hz, 1H), 7.06–7.15 (m, 2H), 7.46 (dd, *J* = 8.97, 4.93 Hz, 2H), 10 9.61 (s, 1H).

#### Step B: Preparation of *tert*-Butyl 2-(((1*r*,4*r*)-4-((4-Fluorophenylcarbamoyloxy)methyl)cyclohexyl)methoxy)acetate

To a solution of methyl 4-fluoro-2-((1*r*,4*r*)-4-(hydroxymethyl)cyclohexyl)phenylcarbamate (2.0 g, 7.11 mmol) and rhodium(II) acetate (0.157 g, 0.355 mmol) in CH_2_Cl_2_ (10 mL) at 0 °C was slowly added *tert*-butyl 2-diazoacetate (1.084 mL, 7.82 mmol) predissolved in CH_2_Cl_2_ (5 mL) via an addition funnel. The reaction was stirred at 0 °C for 1 h and then at room temperature for another 1 h. After removal of the solvent, the residue was purified by silica gel column chromatography to yield the title compound as a tan solid (1.9 g, 61%). LCMS *m*/*z* = 340.4 [M – *tert*-butyl + H]^+^; ^1^H NMR (400 MHz, DMSO-*d*_6_) δ ppm 0.97 (d, *J* = 10.36 Hz, 4H), 1.42 (s, 9H), 1.44–1.53 (m, 1H), 1.54–1.64 (m, 1H), 1.72–1.82 (m, 4H), 3.26 (d, *J* = 6.32 Hz, 2H), 3.90 (d, *J* = 6.57 Hz, 2H), 3.92 (s, 2H), 7.06–7.15 (m, 2H), 7.46 (dd, *J* = 8.84, 4.93 Hz, 2H), 9.61 (s, 1H).

#### Step C: Preparation of 2-(((1*r*,4*r*)-4-((Bis(4-fluorophenyl)carbamoyloxy)methyl)cyclohexyl)methoxy)acetic Acid

The title compound was obtained as a white solid from *tert*-butyl 2-(((1*r*,4*r*)-4-((4-fluorophenylcarbamoyloxy)methyl)cyclohexyl)methoxy)acetate and 1-fluoro-4-iodobenzene, using a similar method to the one described for compound **5e**. LCMS *m*/*z* = 434.5 [M + H]^+^; purity, 99%.

#### Preparation of 2-(((1*r*,4*r*)-4-(((4-Fluorophenyl)(3-fluorophenyl)carbamoyloxy)methyl)cyclohexyl)methoxy)acetic Acid (**5s**)

The title compound was obtained as a white solid from 1-chloro-4-iodobenzene and *tert*-butyl 2-(((1*r*,4*r*)-4-((3-fluorophenylcarbamoyloxy)methyl)cyclohexyl)methoxy)acetate, using a similar method to the one described for compound **5e**. LCMS *m*/*z* = 434.4. [M + H]^+^; purity, 99%.

#### Preparation of 2-(((1*r*,4*r*)-4-(((4-Chloro-3-fluorophenyl)(3-fluorophenyl)carbamoyloxy)methyl)cyclohexyl)methoxy)acetic Acid (**5t**)

The title compound was obtained as a white solid from 1-chloro-2-fluoro-4-iodobenzene and *tert*-butyl 2-(((1*r*,4*r*)-4-((3-fluorophenylcarbamoyloxy)methyl)cyclohexyl)methoxy)acetate, using a similar method to the one described for compound **5e**. LCMS *m*/*z* = 468.5. [M + H]^+^; purity, 99%.

### First Scale-Up Route

#### Preparation of Sodium 2-(((1*r*,4*r*)-4-(((4-Chlorophenyl)(phenyl)carbamoyloxy)methyl)cyclohexyl)methoxy)acetate (**5c**). Step A Preparation of 4-Chlorophenyl(phenyl)carbamic Chloride (**12c**, X = Cl, R^1^ = H, R^2^ = 4-Cl)

4-Chloro-*N*-phenylaniline (10 g, 49.1 mmol) was dissolved in CH_2_Cl_2_ (30 mL). The solution was cooled on an ice bath (0 °C), and then triphosgene (16.03 g, 54.0 mmol) was added. Pyridine (5.56 mL, 68.7 mmol) predissolved in CH_2_Cl_2_ (10 mL) was added slowly via addition funnel to the reaction mixture (exothermic; the solution went from a green color to an orange-yellow color). Upon complete addition, the reaction was stirred on the ice bath for another 15 min and then warmed to room temperature and stirred for an hour. After this time, the reaction was complete as judged by TLC. The reaction was again cooled on an ice bath and quenched by the slow addition of H_2_O (20 mL; exothermic; reaction bubbled vigorously). The reaction was extracted (100 mL each of H_2_O and CH_2_Cl_2_). The aqueous layer was extracted again with CH_2_Cl_2_ (100 mL). The combined organic layers were dried, concentrated, and the product was purified by chromatography (0, 10, 20, 30, 40% CH_2_Cl_2_/hexanes step gradient) to provide 4-chlorophenyl(phenyl)carbamic chloride (12.34 g, 45.9 mmol, 93% yield) as a light yellow oil. ^1^H NMR (400 MHz, DMSO-*d*_6_) δ ppm 7.31–7.81 (m).

#### Step B: Preparation of ((1*r*,4*r*)-4-(Hydroxymethyl)cyclohexyl)methyl 4-Chlorophenyl(phenyl)carbamate (**11**, R^1^ = H, R^2^ = 4-Cl)

4-Chlorophenyl(phenyl)carbamic chloride (12.34 g, 46.4 mmol) and (1*r*,4*r*)-cyclohexane-1,4-diyldimethanol (6.69 g, 46.4 mmol) were dissolved in pyridine (50 mL, 618 mmol). The reaction mixture was heated to reflux overnight and then cooled and concentrated under reduced pressure. The residue was resuspended in Et_2_O/EtOAc (50:50), filtered and the filtered solid washed with Et_2_O/EtOAc (50:50). The filtrate was partitioned between 1 M HCl (200 mL) and EtOAc (200 mL). The aqueous layer was extracted again with EtOAc (100 mL). The organic layers were combined and washed with H_2_O (200 mL), dried, and concentrated. The residue was purified by silica gel column chromatography to provide the title compound as a light pink colored solid (10.4 g, 60%). LCMS *m*/*z* = 374.1 [M + H]^+^; ^1^H NMR (400 MHz, DMSO-*d*_6_) δ ppm 0.73–0.92 (m, 4H), 1.13–1.27 (m, 1H), 1.36-l.48 (m, 1H), 1.53–1.62 (m, 2H), 1.62–1.73 (m, 2H),3.17 (d, *J* = 6.19 Hz, 2H), 3.89 (d, *J* = 6.06 Hz, 2H), 4.29 (br s, 1H), 7.23–7.32 (m, 5H), 7.34–7.45 (m, 4H).

#### Step C: Preparation of *tert*-Butyl 2-(((1*r*,4*r*)-4-(((4-Chlorophenyl)(phenyl)carbamoyloxy)methyl)cyclohexyl)methoxy)acetate (**9**, R = ^*t*^Bu, R^1^ = H, R^2^ = 4-Cl)

((1*r*,4*r*)-4-(Hydroxymethyl)cyclohexyl)methyl4-chlorophenyl(phenyl)carbamate (8.9 g, 23.80 mmol) was dissolved in CH_2_Cl_2_ (30 mL). Diacetoxyrhodium (0.526 g, 1.190 mmol) was added, and the reaction was cooled on an ice bath. *tert*-Butyl 2-diazoacetate (3.63 mL, 26.2 mmol) predissolved in CH_2_Cl_2_ (10 mL) was added slowly to the reaction via an addition funnel. The reaction was stirred in an ice bath for 1 h, warmed to room temperature, and stirred for an additional 1 h. After removal of the solvent, the residue was purified by silica gel column chromatography to provide the title compound as a colorless oil (8.8 g, 75%). LCMS *m*/*z* = 432.6 [M – *tert*-butyl group + H]^+^; ^1^H NMR (400 MHz, DMSO-*d*_6_) δ ppm 0.77–0.95 (m, 4H), 1.33–1.50 (m, 2H), 1.42 (s, 9H), 1.52–1.62 (m, 2H), 1.63–1.75 (m, 2H), 3.22 (d, *J* = 6.32 Hz, 2H), 3.83–3.93 (m, 4H), 7.23–7.32 (rn, 5H), 7.35–7.44 (m, 4H).

#### Step D: Preparation of Sodium 2-(((1*r*,4*r*)-4-(((4-Chlorophenyl)(phenyl)carbamoyloxy)methyl)cyclohexyl)methoxy)acetate (**5c**, Sodium Salt)

*tert*-Butyl 2-(((1*r*,4*r*)-4-(((4-chlorophenyl)(phenyl)carbamoyloxy)methyl)cyclohexyl)methoxy)acetate (8.8 g, 18.03 mmol) was dissolved in HCl (4 M in dioxane, 100 mL, 400 mmol). The reaction was stirred at room temperature overnight and concentrated under reduced pressure to provide an oil. The oil was partitioned between H_2_O (100 mL) and EtOAc (100 mL). The aqueous layer was extracted again with EtOAc (100 mL). The combined organic layer was washed with H_2_O (150 mL), dried, and concentrated to yield a light yellow oil. The oil was dissolved in a minimal amount of MeOH (10–20 mL) and cooled in an ice bath. NaOH (1M, 27.0 mL, 27.0 mmol) was added with stirring during which time a white solid precipitate was formed. The mixture was diluted with H_2_O (20 mL). The solid was collected by filtration and washed with cold H_2_O (20 mL). The resulting white solid was dried in a vacuum oven (60 °C overnight) to provide the title compound (7.7 g, 98%). LCMS *m*/*z* = 432.5 [M + H]^+^; purity, 99%; ^1^H NMR (400 MHz, DMSO-*d*_6_) δ ppm 0.73–0.93 (m, 4H), 1.28–1.40 (m, 1H), 1.40–1.50 (m, 1H), 1.50–1.61 (m, 2H), 1.63–1.77 (m, 2H), 3.16 (d, *J* = 6.57 Hz, 2H), 3.47 (s, 2H), 3.89 (d, *J* = 6.06 Hz, 2H), 7.23–7.32 (m, 5H), 7.35–7.44 (m, 4H).

#### Preparation of Sodium 2-(((1*r*,4*r*)-4-(((3-Fluorophenyl)(phenyl)carbamoyloxy)methyl)cyclohexyl)methoxy)acetate (**5g**). Step A: Preparation of 3-Fluoro-*N*-phenylaniline (**11**, R^1^ = H, R^2^ = 3-F)

In a 3 L, three-neck flask equipped with mechanical stirring, a solution of 3-fluoroaniline (75 g, 675 mmol), bromobenzene (73 mL, 690 mmol), and dichloro[1,1′-20-bis(diphenylphosphino)ferrocene]palladium(II) dichloromethane adduct (15 g, 18 mmol) in anhydrous toluene (1.3 L) containing sodium *tert*-butoxide (130 g, 1.35 mol) was heated at 105 °C for 3 h. The reaction mixture was then cooled to 80 °C and quenched by gradually pouring the reaction mixture into ice/water (1 L). The aqueous layer was removed and was then extracted with an additional volume of toluene (300 mL). The organic extracts were combined, rinsed with brine, dried over MgSO_4_, and passed through a silica plug (1.3 kg), eluting with toluene. The solvent was removed to give a dark amber oil (86 g, 68%). LCMS *m*/*z* = 188 [M + H]^+^; ^1^H NMR (400 MHz, CDCl_3_) δ 6.45 (t, *J* = 8.5 Hz, 1H), 6.62–6.66 (m, 2H), 6.87 (t, *J* = 7.2 Hz, 1H), 6.98 (d, *J* = 7.6 Hz, 2H), 7.05 (q, *J* = 7.5 Hz, 1H), 7.17 (t, *J* = 8.6 Hz, 2H).

#### Step B: Preparation of 3-Fluorophenyl(phenyl)carbamic Chloride (**12**, X = Cl, R^1^ = H, R^2^ = 3-F)

A 3 L three-neck mechanically stirred flask under N_2_ containing a solution of 3-fluoro-*N*-phenylaniline (86 g, 460 mmol) in 1.2 L of dichloromethane was cooled in an ice bath to 0 °C, and then triphosgene (150 g, 505 mmol) was added. A solution of pyridine (52 mL, 640 mmol) in dichloromethane (200 mL) was added in a dropwise fashion. Initial addition resulted in a temperature spike to 25 °C after the first 10 mL had been added over 10 min. The addition was paused, and the reaction mixture was stirred for 1 h while recooling to 5 °C. Addition of the pyridine solution was again commenced at a rate of 5 mL/min, at which an internal reaction temperature of 5–10 °C could be maintained. After addition was complete (about 1 h), the reaction had proceeded to completion and was quenched by the slow addition of ice–water (500 g). Gas was passed through a 20% sodium hydroxide trap until all gas evolution had ceased (about 3 h). The aqueous layer was removed and was then extracted with an additional 300 mL of dichloromethane. The organic extracts were combined, dried over MgSO_4_, and the solvent was removed. Clean product was readily isolated as a viscous, pink oil, which gradually formed a pale pink solid upon seeding with crystals. LCMS *m*/*z* = 250.0 [M + H]^+^; ^1^H NMR (CDCl_3_, 400 MHz) δ 7.00–7.07 (m, 1H), 7.10 (d, *J* = 9.6 Hz, 1H), 7.15 (d, *J* = 8.1 Hz, 1H), 7.35 (d, *J* = 7.7 Hz, 2H), 7.35–7.41 (m, 2H), 7.42–7.48 (m, 2H).

#### Step C: Preparation of 4-(Dimethylamino)-1-((3-fluorophenyl)(phenyl)carbamoyl)pyridinium Chloride (**12**, X = DMAP, R^1^ = H, R^2^ = 3-F)

To a solution of crude 3-fluorophenyl(phenyl)carbamic chloride (62.4 g, 250 mmol) in acetonitrile (500 mL) in a 2 L mechanically stirred three-neck flask was added a solution of 4-dimethylaminopyridine (30.5 g, 250 mmol) in 500 mL acetonitrile. The flask warmed slightly as crystallization began to occur and then cooled again to ambient temperature. The resulting suspension was stirred overnight, cooled to 10 °C in an ice bath and the precipitate filtered, rinsing with cold acetonitrile (100 mL) to provide the title compound as a fine, white solid (88.27 g). LCMS *m*/*z* = 336. [M + H]^+^; ^1^H NMR (400 MHz, methanol-*d*_4_) δ 3.29 (s, 6H), 6.92 (d, *J* = 8.1 Hz, 2H), 7.11 (t, *J* = 8.6 Hz, lH), 7.16 (d, *J* = 8.8 Hz, lH), 7.21 (d, *J* = 9.5 Hz, lH), 7.33–7.38 (m, 3H), 7.41–7.47 (m, 3H), 8.37 (d, *J* = 8.1 Hz, 2H).

#### Step D: Preparation of ((1*r*,4*r*)-4-(Hydroxymethyl)cyclohexyl)methyl-3-fluorophenyl(phenyl)carbamate (**13**, R^1^ = H, R^2^ = 3-F)

A suspension of 4-(dimethylamino)-1-((3-fluorophenyl)(phenyl)carbamoyl)pyridinium chloride (88.25 g, 237 mmol), (1*r*,4*r*)-cyclohexane-1,4-diyldimethanol (137 g, 950 mmol), and 4-dimethylaminopyridine (29.0 g, 237 mmol) in acetonitrile (1 L) was heated at 53 °C for 18 h. Upon cooling, the solvent was removed, and the residue was taken up in isopropyl acetate (500 mL) and 1 M HCl (500 mL), heated to suspend all solids, and then filtered through glass fiber filter paper to attempt to remove a sparingly soluble bis-carbamate impurity. The aqueous filtrate was discarded, and the organic filtrate was washed with an additional 500 mL of 1 N HCl, followed by water (5 × 500 mL). Heptane (100 mL) was added to the organic phase, which was further washed with water (2 × 500 mL) and brine (100 mL), dried over MgSO_4_, and concentrated to dryness. The residue was taken up in isopropyl acetate (100 mL), and heptane (300 mL) was added. Crystals gradually formed over 1 h, providing a white precipitate, which was collected by filtration, and washed with 25% isopropyl acetate/heptane (100 mL). The filtrate was concentrated to dryness, and the residue was taken up in hot 25% isopropyl acetate/heptane (100 mL) and filtered hot. As the filtrate cooled, more solids precipitated, which were collected by filtration and combined with the first crop. This material still contained about 5% bis-carbamate byproduct, which could not be readily removed by filtration. The solid was then taken up in dichloromethane (200 mL) and subjected to plug filtration over 1.6 kg of silica gel, eluting the product with 20% ethyl acetate/dichloromethane to provide the title compound as a white solid (71 g, 83%) and the remaining bis-carbamate with dichloromethane. LCMS *m*/*z* = 358. [M + H]^+^; ^1^H NMR (CDCl_3_, 400 MHz) δ 0.91–0.98 (m, 4H), 1.35–1.44 (m, 1H), 1.54–1.60 (m, 1H), 1.68–1.73 (m, 2H), 1.79–1.83 (m, 2H), 3.45 (d, *J* = 6.4 Hz, 2H), 4.01 (d, *J* = 6.4 Hz, 2H), 6.91 (t, *J* = 7.6 Hz, 1H), 7.04 (d, *J* = 8.6 Hz, 2H), 7.22–7.30 (m, 4H), 7.38 (t, *J* = 7.8 Hz, 2H).

#### Step E: Preparation of Ethyl 2-(((1*r*,4*r*)-4-(((3-Fluorophenyl)phenyl)carbamoyloxy)methyl)cyclohexyl)methoxy)acetate (**7**, R = Et, R^1^ = H, R^2^ = 3-F)

In a 250 mL three-neck reactor equipped with a stirrer, a thermocouple, a cooling bath, an addition funnel, and a nitrogen inlet was placed ((1*r*,4*r*)-4-(hydroxymethyl)cyclohexyl)methyl-3-fluorophenyl(phenyl)carbamate (8 g, 22.38 mmol). This was dissolved in dichloromethane (150 mL). The mixture was cooled and stirred well at 4 °C in an isopropanol/ice bath. Diacetoxyrhodium (0.5 g, 1.12 mmol) was added. After the addition was complete, ethyl diazoacetate (3.69 g, 32.34 mmol) was dissolved in dichloromethane (30 mL) and added to the reaction mixture keeping the temperature below 10 °C. After addition, the reaction mixture was warmed to 30 °C and the progress of the reaction was followed by LCMS. On the basis of the LCMS in-process control, further batches of ethyl diazoacetate (0.63 g, 5.52 mmol, followed by 0.710 g, 6.22 mmol dissolved in dichloromethane (15 mL)) were added separately at 25 °C. The reaction mixture was stirred at 30 °C until LCMS showed complete consumption of the starting material. The reaction mixture was diluted with water (100 mL), and the mixture was filtered through a bed of Celite (35 g) to remove the catalyst. The organic layer was then separated and dried over magnesium sulfate (15 g) and filtered. The solvent was removed to provide the title compound as an oil (9.9 g), which still contained a small amount of ethyl diazoacetate and was used without further purification. LCMS *m*/*z* = 444.5 [M + H]^+^; ^1^H NMR (400 MHz, DMSO-*d*_6_) δ ppm 0.82–0.96 (m, 4H), 1.22 (t, *J* = 7.07 Hz, 3H), 1.27 (t, *J* = 7.14 Hz, 1H), 1.37–1.53 (m, 2H), 1.57–1.78 (m, 4H), 3.26 (d, *J* = 6.32 Hz, 2H), 3.94 (d, *J* = 6.06 Hz, 2H), 4.06 (s, 2H), 4.14 (q, *J* = 7.07 Hz, 3H), 4.23 (q, *J* = 7.07 Hz, 1H), 7.05–7.11 (m, 2H), 7.24 (dt, *J* = 10.64, 2.26 Hz, 1H), 7.28–7.35 (m, 3H), 7.36–7.45 (m, 3H).

#### Step F: Preparation of 2-(((1*r*,4*r*)-4-(((3-Fluorophenyl)(phenyl)carbamoyloxy)methyl)cyclohexyl)methoxy)acetic Acid (**5g**, Free Acid)

In a 500 mL, three-neck reactor equipped with a stirrer, a thermocouple, a heating oil bath, an addition funnel, and a nitrogen inlet was placed ethyl 2-(((1*r*,4*r*)-4-(((3-fluorophenyl)(phenyl)carbamoyloxy)methyl)cyclohexyl)methoxy)acetate (9.9 g, 22.32 mmol), which was dissolved in acetonitrile (150 mL). To this mixture lithium bromide (19.58 g, 225.00 mmol) was added. After the addition was complete, triethylamine (6.84 g, 67.6 mmol) was added and the reaction mixture was heated at 70 °C. The progress of the reaction was followed by LCMS. On the basis of the LCMS, the starting material was consumed in 2 h. Solvent was removed, and the reaction mixture was diluted with water (200 mL) and made acidic with hydrochloric acid (3 M, 7.8 mL). The precipitated solids were filtered, and the wet solid was dissolved in isopropyl acetate (200 mL). The isopropyl acetate solution was dried over magnesium sulfate (15 g), filtered, and the solvent was removed. The residue was dried in a vacuum oven to provide the title compound (9.2 g, 95%). LCMS *m*/*z* = 416.4 [M + H]^+^; ^1^H NMR (400 MHz, DMSO-*d*_6_) δ ppm 0.81–0.96 (m, 4H), 1.36–1.53 (m, 2H), 1.55–1.77 (m, 4H), 3.25 (d, *J* = 6.44 Hz, 2H), 3.93 (d, *J* = 5.94 Hz, 2H), 3.97 (s, 2H), 7.05–7.13 (m, 2H), 7.24 (dt, *J* = 10.64, 2.26 Hz, 1H), 7.28–7.36 (m, 3H), 7.37–7.46 (m, 3H), 12.53 (br s, 1 H)

#### Step G: Preparation of 2-(((1*r*,4*r*)-4-(((3-Fluorophenyl)(phenyl)carbamoyloxy)methyl)cyclohexyl)methoxy)acetic Acid Sodium Salt (**5g**, Sodium Salt)

In a 500 mL, three-neck reactor equipped with a stirrer, a thermocouple, a heating oil bath, an addition funnel, and a nitrogen inlet was placed 2-(((1*r*,4*r*)-4-(((3-fluorophenyl)(phenyl)carbamoyloxy)methyl)cyclohexyl)methoxy)acetic acid (9.2 g, 22.83 mmol) and 2-propanol (100 mL). The reaction mixture was heated at 30 °C (bath temperature) until all of the acid was dissolved completely. To the orange solution, sodium hydroxide (1 M, 22 mL, 22 mmol) was added slowly keeping the internal temperature around 25 °C. The sodium salt separated out as crystals. The thick slurry was stirred at 25 °C for 2 h and then cooled in an ice–water bath for 20–40 min. The solids were filtered and dried in a vacuum oven at 40 °C overnight until most of the residual 2-propanol was removed to provide the title compound (7.4 g, 74%). LCMS *m*/*z* = 416.5 [M + H]^+^; purity, 99%. ^1^H NMR (400 MHz, DMSO-*d*_6_) δ ppm 0.77–0.95 (m, 4H), 1.34–1.53 (m, 2H), 1.55–1.75 (m, 4H), 3.19 (d, *J* = 6.44 Hz, 2H), 3.52 (s, 2H), 3.93 (d, *J* = 5.94 Hz, 2H), 7.05–7.13 (m, 2H), 7.24 (dt, *J* = 10.64, 2.26 Hz, 1H), 7.28–7.35 (m, 3H), 7.37–7.46 (m, 3H).

### Second Scale-Up Route

#### Step A: Preparation of ((1*r*,4*r*)-4-(Hydroxymethyl)cyclohexyl)methyl 4-chlorophenyl(phenyl)carbamate (**13** R^1^ = H, R^2^ = 4-Cl)

A solution of (1*r*,4*r*)-cyclohexane-1,4-diyldimethanol in acetonitrile was prepared by charging (1*r*,4*r*)-cyclohexane-1,4-diyldimethanol (4.22 kg) and acetonitrile (13.37 kg) to a 50 L glass lined reactor equipped with overhead agitation, jacket temperature control, and a nitrogen inlet. The reactor contents were stirred at 163 rpm and heated to 65 °C for 1 h to dissolve most of the (1*r*,4*r*)-cyclohexane-1,4-diyldimethanol. The mixture was cooled to 45 °C and transferred to a 20 L fluorinated high density polyethylene (HDPE) carboy for addition to **12** (X = CDI, R^1^ = H, R^2^ = 4-Cl) at a later time.

**11** (R^1^ = H, R^2^ = 4-Cl, 1.71 kg), K_3_PO_4_ (0.53 kg), carbonyl diimidazole (CDI, 1.49 kg), and acetonitrile (6.69 kg) were charged to a 50 L glass lined reactor equipped with overhead agitation, jacket temperature control, and a nitrogen inlet. The reactor contents were stirred at 175 rpm and heated to 65–70 °C for 4.5 h, after which conversion of **11** (R^1^ = H, R^2^ = 4-Cl) to **12** (R^1^ = H, R^2^ = 4-Cl) was verified to be greater than 98.0% by HPLC peak area. The reaction mixture was cooled to less than 40 °C and the (1*r*,4*r*)-cyclohexane-1,4-diyldimethanol solution prepared earlier added to the mixture. The reactor contents were stirred at 178 rpm and heated at 65–70 °C for 5 h, after which conversion was 99.6% by HPLC peak area

The 50 L reactor contents were filtered, and the filter cake was rinsed with acetonitrile (1.10 kg). The filtrate was transferred back to the reactor. Most of the acetonitrile (18.48 kg) was then removed at an internal temperature of less than 40 °C by vacuum distillation at 80 mmHg. Water (5.67 kg) was added to the 50 L reactor, and approximately 1.55 kg of water/acetonitrile mixture was then removed by vacuum distillation at an internal temperature of less than 40 °C and 70 mmHg.

Further water (5.68 kg) was then added to the 50 L reactor, and the product precipitated during the addition. The resulting mixture was stirred at 20–25 °C for 4 h. The precipitated product was filtered and washed in two portions with aqueous acetonitrile (1.68 kg of acetonitrile dissolved in 6.39 kg of water). The product was vacuum-dried at ≤60 °C to a loss on drying value of ≤2.0 wt %. **13** (R^1^ = H, R^2^ = 4-Cl, 2.29 kg) was thus obtained in 73% yield (over 2 telescoped steps) and 98.1% purity by HPLC peak area.

#### Step B: Preparation of Sodium 2-(((1*r*,4*r*)-4-(((4-Chlorophenyl)(phenyl)carbamoyloxy)methyl)cyclohexyl)methoxy)acetate (**5c**)

**13** (R^1^ = H, R^2^ = 4-Cl, 1.70 kg), tetrabutylammonium bromide (0.45 kg), and toluene (8.82 kg) were charged to a 50 L glass lined reactor equipped with overhead agitation, jacket temperature control, and a nitrogen inlet. After **13** (R^1^ = H, R^2^ = 4-Cl) had dissolved by stirring at 20 °C for 1 h, *tert*-butyl bromoacetate (1.33 kg) was added at 20 °C to the reaction mixture. The jacket temperature was set to −5 °C, and 50 wt % aq sodium hydroxide (15.31 kg) was added sufficiently slowly to maintain the stirred reaction mixture at 2–10 °C with reactor jacket cooling. The mixture was stirred at that temperature for 7.2 h. Conversion of **13** (R^1^ = H, R^2^ = 4-Cl) to **9** (R^1^ = H, R^2^ = 4-Cl) was then verified to be 93.6% by HPLC peak area.

Thereafter, the reactor contents were heated at 45–51 °C for 3.0 h. Conversion of **9** (R = ^*t*^Bu, R^1^ = H, R^2^ = 4-Cl) to **5c** was then verified to be 99.8% by HPLC peak area. The reactor contents were then cooled to 42 °C, and hydrochloric acid, conc (20.04 kg), was added to the mixture slowly so as to maintain an internal temperature at ≤45 °C. The mixture was filtered to remove the solid sodium chloride from the reactor, and the filtrate was collected in clean carboys.

After cleaning the reactor with water, filtrate from the carboys was transferred back to the reactor and the phases separated. The aqueous layer was extracted with toluene (4.43 kg), and the phases separated again. The toluene layers were combined and the mixture was vacuum distilled at 42 °C and 5 mmHg to remove toluene (7.5 kg). IPA (13.36 kg) was charged to the reactor, and the resulting solution was vacuum distilled at 31.4 °C and 5.7 mmHg to remove solvent (16.98 kg). Acetone (11.10 kg) and water (3.08 kg) were charged to the reactor and the reactor contents stirred at 20 °C. Thereafter, 12.5% sodium hydroxide (1.25 kg; made by diluting 50 wt % sodium hydroxide with water) was added to the reactor contents to a pH of 9 to 10. The mixture was agitated at 173 rpm for 1 h at 0 °C. The resulting precipitated product was filtered, and the filter cake was washed with acetone (5.58 kg).

The filter cake was transferred to the reactor using acetone (12.59 kg) and water (4.56 kg), and the mixture was heated at 50 °C for 1.5 h. The resulting mixture was filtered through a sintered glass filter and the filtrate transferred to the clean reactor. Acetone (3.23 kg) was added, and the mixture was stirred for 15.7 h at 1.9 °C. The reactor contents were filtered, and the filter cake was washed with acetone (4.00 kg). The filter cake was then transferred back to the reactor with the aid of water (17.01 kg), and 2 N hydrochloric acid (0.90 kg; made by diluting concentrated HCl with water) was added to the reactor contents to a pH of 2. The reactor contents were stirred at 150 rpm and 16 °C for 23.5 h.

The product slurry was collected by filtration, washed with two portions of water (25.54 kg total), dried at 65–70 °C under vacuum for 72.1 h, and finally sieved through a 1.18 mesh screen. **5c** (1.16 kg) was thus obtained in 59% yield (over two telescoped steps) and in 99.5% purity by HPLC peak area.

#### Preparation of 2-(2-(((1*r*,4*r*)-4-(((4-Chlorophenyl)(phenyl)carbamoyloxy)methyl)cyclohexyl)methoxy)acetamido)ethanesulfonic Acid (**M1**)

**5c** (sodium salt, 10 g, 23.15 mmol) was dissolved in thionyl chloride (40 mL) and the mixture heated at 70 °C for 3 h. The excess thionyl chloride was removed under reduced pressure, and the residual orange solids were dissolved in Ddioxane (100 mL). Taurine (2.9 g, 23.15 mmol) was dissolved in 20% sodium hydroxide (25 mL), cooled to 0 °C, and treated dropwise with the dioxane solution of the acid chloride while keeping the internal temperature of the mixture below 5 °C during addition. When addition was complete, the mixture was allowed to warm to room temperature and then stirred overnight.

LC/MS indicated formation of the title compound and some unreacted taurine and **3c**. The crude mixture was concentrated to remove dioxane, the resulting yellow solid was cooled, and a solution of 1 N HCl (150 mL) was added. The fine solids were filtered (very slow filtration) and washed with 1 N HCl (950 mL). The solids were dissolved in ethanol and concentrated to remove residual water. More ethanol (150 mL) was added and the resultant suspension filtered to remove the excess taurine. The solids were suspended in acetonitrile (200 mL) and stirred overnight. The mixture was then filtered and the resultant solid product dried in a vacuum oven at 50 °C overnight to provide a pale yellow solid (9.62g, 77%). HPLC analysis showed the product to consist of >97% of the title compound and 1.2% of **5c** as well as some other unidentified minor impurities.

In order to fully evaluate the receptor properties of M1 without any contamination with the known active compound **5c**, a small sample was purified by preparative HPLC. Thus, the solid (20 mg) was dissolved in water and purified by preparative RP-HPLC (solvent A, 0.05% TFA; solvent B, 0.05% TFA in acetonitrile, gradient 15–90% B in 20 min, flow rate 20 mL/min, column Alltech Prevail C18, 5 μm, 22 mm × 100 mm, autosampler/fraction collector Gilson 215; HPLC, Shimadzu SCL-10 Avp controller, LC-8ADvp pumps; mass spectrometer, Sciex API 150 EX) with mass-triggered fraction collection and the pure product containing fractions lyophilized. The resulting product M1 (∼9 mg) contained less than 0.06% **5c** by HPLC. Exact mass calcd for C_25_H_31_ClN_2_O_7_S, 538.2; found, LCMS *m*/*z* 539.2 [M + H]^+^; ^1^H NMR (400 MHz, DMSO-*d*_6_) δ (ppm) 0.78–0.97 (m, 4H), 1.37–1.52 (m, 2H), 1.52–1.62 (m, 2H), 1.67–1.77 (m, 2H), 2.54 (t, *J* = 6.44 Hz, 2H), 3.21 (d, *J* = 6.32 Hz, 2H), 3.37 (q, *J* = 5.81 Hz, 2H)3.76 (s, 2H) 3.90 (d, *J* = 6.06 Hz, 2H) 7.22–7.34 (m, 5H), 7.35–7.48)(m, 4H), 7.91 (br s, 1H).

### Receptor Functional Assays

For development of cAMP accumulation assays, recombinant IP or DP1 receptors were stably expressed in CHO-K1 cells and clonal cell lines derived following standard protocols. Receptor expression levels were minimized to preclude receptor reserve effects. HTRF cAMP assays were performed according to the manufacturer’s instructions (Cisbio, cAMP Dynamic 2 assay kit; no. 62AM4PEJ). IP receptor-expressing cells were harvested, resuspended in assay buffer (PBS containing 1 mM IBMX and 0.2% BSA) at a density of 200 000 cells per mL, and dispensed into 384-well assay plates (PerkinElmer Proxiplate no. 6008280) at 5 μL per well. Test compounds were solubilized and serially diluted in DMSO using 5-fold dilutions to generate a 10-point dose–response curve with a top concentration of 10 μM. The samples were then further diluted in PBS to achieve a 2× stock. Diluted compounds were then transferred to a triplicate set of assay plates (5 μL per well). After a 1 h incubation at room temperature, 5 μL of cAMP-D2 reagent diluted in lysis buffer was added to each well followed by 5 μL of cryptate reagent. Plates were then incubated at room temperature for 1 h prior to reading. Time-resolved fluorescence measurements were collected on PerkinElmer Envision or BMG Pherastar microplate readers.

For the EP_3v6_ receptor functional assay, melanophores were transfected by electroporation using 20 μg of recombinant human EP_3v6_ receptor plasmid DNA per 400 μL of cell suspension. Transfected cells were immediately resuspended in fresh growth medium and plated in 384-well clear, polystyrene microplates. Plated cells were incubated at 27 °C for 48 h after transfection in order to achieve optimal receptor expression. To perform the assay, growth medium was removed from the assay plates and replaced with assay buffer (40 μL/well, 0.7× PBS, pH 7.3, supplemented with 20 nM melatonin to induce pigment aggregation). Following a 90 min incubation at room temperature, a 650 nm absorbance reading was collected and test compounds (10 μL per well) were transferred to the assay plates. Plates were incubated for 90 min at room temperature to allow compound-induced pigment redistribution to take place, and final absorbance reading was then collected. Test compounds were solubilized and serially diluted in DMSO, using 5-fold dilutions, to generate a 10-point dose–response curve with a top concentration of 10 μM (final assay concentration). Samples were then further diluted in assay buffer to achieve a 5× stock prior to addition to the assay plates. A SpectraMax absorbance plate reader (Molecular Devices, Inc.) was used for data collection.

### Radioligand Binding Assays

#### Membrane Preparation

CHO-K1 cells expressing recombinant IP receptors were harvested, washed with ice-cold phosphate buffered saline, pH 7.4 (PBS), and then centrifuged at 48 000*g* for 20 min at 4 °C. The resulting cell pellet was then resuspended in ice-cold PBS containing 20 mM HEPES, pH 7.4, and 0.1 mM EDTA, homogenized on ice using a Brinkman Polytron, and centrifuged (48 000*g* for 20 min at 4 °C). This resuspension and centrifugation process was repeated one further time. Crude membrane pellets were stored at −80 °C until used for radioligand binding assays.

#### Radioligand Binding Assay Protocol

Radioligand binding assays were conducted using the IP receptor agonist [^3^H]-iloprost as radioligand, and nonspecific binding was determined in the presence of unlabeled 10 μM iloprost. Specific assay conditions for each receptor are listed in Table 5. Competition experiments consisted of addition of 45 μL of assay buffer (PBS containing 20 mM HEPES and 10 mM MgCl_2_, pH 7.4), 100 μL of membranes, 50 μL of [^3^H]-iloprost, and 5 μL of test compound diluted in assay buffer to 96-well microtiter plates, which were then incubated for 1 h at room temperature. Assay incubations were terminated by rapid filtration through PerkinElmer GF/C filtration plates pretreated with 0.3% PEI, under vacuum pressure using a 96-well Packard filtration apparatus, followed by multiple washes with ice-cold wash buffer (PBS containing 20 mM HEPES and 0.1 mM EDTA, pH 7.4). Plates were then dried at 45 °C for a minimum of 2 h. Finally, 25 μL of BetaScint scintillation cocktail was added to each well and plates were counted in a Packard TopCount scintillation counter. In each competition study, test compounds were dosed at 10 concentrations with triplicate determinations at each test concentration. A reference compound, typically iloprost, was included in every runset for quality control purposes.

**Table 5:** Assay Conditions for Recombinant IP Receptor Binding Assay

receptor	[^3^H]-iloprost assay concentration (nM)	membrane protein (μg)	measured *K*_d_ (nM)	literature *K*_d_ (nM)
human IP	7.57	20	7.0	9.82
monkey IP	7.57	20	11.9	NA
dog IP	7.57	20	15.4	NA
rat IP	7.57	20	7.0	NA

Additional (non-IP) receptor binding assays were carried out by MDS Pharma (Taiwan) and CEREP (France).

### In Vivo Assay

All in vivo experiments were performed in accordance with the ACS ethical guidelines

### Animal Model

In the study to evaluate the effects of oral administration of **5c** on MCT-induced PAH, we used 38 male Sprague–Dawley rats weighing 200–250 g; 30 rats were chosen randomly to receive a subcutaneous injection of 60 mg/kg MCT on day 1 of the trial, while the remaining 8 received a subcutaneous injection of saline (sham, *n* = 8). The MCT-injected rats were then assigned to one of three treatment protocols: oral treatment with **5c** from day 1 (10 and 30 mg/kg, *n* = 10 in each dose group) and oral 20% HPBCD in saline (control group, *n* = 10).

### In Vivo Experimental Protocol

Following anesthesia by intraperitoneal injection of 30 mg/kg pentobarbital, rats were given a subcutaneous injection of either 60 mg/kg MCT (Sigma, catalog no. C2401-1G) or 0.9% saline for the sham group. The following day and for 20 days thereafter, 10 mg/kg and 30 mg/kg (**5c** treatment groups, *n* = 10) or oral 20% HPBCD in saline (control group, *n* = 10) was administered twice daily by oral gavage. Hemodynamic measurements and histologic analyses were performed on day 21; this time point was based on survival curve analyses (2 animals that died on study in the control group on days 19 and 20 could not be evaluated; hence only 8 animals are included in the measurements for this group and not 10). Hemodynamic measurements were performed with animals from the 30 mg/kg group (but not the 10 mg/kg group) anesthetized with 60 mg/kg pentobarbital. Under anesthesia, each animal was intubated with a rodent ventilator (Harvard Apparatus, model 683) at 70 breaths/min of a rate and a tidal volume of 2.5 mL in supine position on a heating pad set at 37 °C. A small opening in the apex of the right ventricle was made using a 23G needle, then a Millar catheter (Millar Instruments, Inc. model SPR-320) was placed into the right ventricle through the opening in the apex, then into the pulmonary artery (PA) for the pressure measurement. A second catheter was inserted through the right jugular vein into the right ventricle (RV) for the measurement of mean arterial pressure. After stabilization, heart rate, mean arterial pressure, and systolic RV pressure were calculated from 20 consecutive heart beats in each rat. Due to the technical difficulty involved in the catheter placement, 5 animals in the 30 mg/kg treated group could not be stabilized sufficiently for measurements to be made; hence a data set of only 5 animals in the high dose group was used for the mPAP figure.

The ventricles and lungs were excised and weighed. The ratio of RV weight to left ventricle plus septum (LV + S) was calculated as an index of right ventricular hypertrophy.

In a separate experiment, the same protocol was used with **5g** except that doses of 15 and 30 mg/kg were used as a result of the weak effect of **5c** on (RV/(LV + S)) at 10 mg/kg in the first experiment.

### Morphometric Analysis of Pulmonary Arterioles

As the collection and preservation of the tissues are very resource intensive, 5 animals were selected from each group for measurement of vessel thickness; this was sufficient to obtain clear statistical differences between groups. Paraffin sections of 4 μm thickness were obtained from the lower region of the right lung and stained with hematoxylin/eosin. Analysis of the medial wall thickness of the pulmonary arterioles was performed. In brief, the external diameter and medial wall thickness were measured in 20 muscular arteries (25–100 μm external diameters) per lung section. For each artery, the medial wall thickness was expressed as follows: % wall thickness = [(external diameter – internal diameter)/external diameter] × 100. Each vessel was measured twice, perpendicularly along the long axis and short axis, and averages of both measurements were used.

All data are expressed as mean ± SEM. Comparisons of parameters among the three groups were made by one-way analysis of variance (ANOVA), followed by Newman–Keuls’ test. Comparisons of the time course of parameters between two groups were made by two-way ANOVA for repeated measures, followed by Newman–Keuls’ test. A value of*P* < 0.05 was considered statistically significant. Survival curves were derived by the Kaplan–Meier method and compared by log-rank test. *P* < 0.05 was considered statistically significant.

## Supporting Information Available

Detailed procedures and summary data for additional in vitro assays and further data from in vivo assays for compound **5c**; DMPK profile in the cynomolgus monkey for **5c**; physical form characterization data for **5c** and **5g**; receptor binding profile of **5c** (PDF)

## Abbreviations Used

ADP: adenosine 5′-diphosphate
AUC: area under the curve
BDC: bile-duct cannulated
Boc: *tert*-butyloxycarbonyl
cAMP: 3′,5′-cyclic adenosine monophosphate
cGMP: 3′,5′-cyclic guanosine monophosphate
CHO: Chinese hamster ovary
Cl: clearance
clogP: calculated log *P* value
*C*_max_: maximum plasma concentration
CYP: cytochrome P450
DCM: dichloromethane
DMAP: 4-dimethylaminopyridine
DMPK: drug metabolism and pharmacokinetics
*F*(sp^3^): fraction of sp^3^ carbon atoms
GPCR: G-protein-coupled receptor
hERG: human-ether-a-go-go-related gene
IND: investigational new drug
mp: melting point
PAH: pulmonary arterial hypertension
PDE5: phosphodiesterase-5
PK: pharmacokinetics
SAR: structure–activity relationship
USAN: United States Adopted Names

## References

[ref1] Rubanyi G. M. The role of endothelium in cardiovascular homeostasis and diseases. J. Cardiovasc. Pharmacol. 1993, 22 (Suppl. 4), S1–S14. 10.1097/00005344-199322004-00002. 7523767

[ref2] Christman B. W.; McPherson C. D.; Newman J. H.; King G. A.; Bernard G. R.; Groves B. M.; Loyd J. E. An imbalance between the excretion of thromboxane and prostacyclin metabolites in pulmonary hypertension. N. Engl. J. Med. 1992, 327, 70–75. 10.1056/NEJM199207093270202. 1603138

[ref3] Rich S. Clinical insights into the pathogenesis of primary pulmonary hypertension. Chest 1998, 114, 237S–241S. 10.1378/chest.114.3_Supplement.237S. 9741575

[ref4] Rubin L. J.; Groves B. M.; Reeves J. T.; Frosolono M.; Handel F.; Cato A. E. Prostacyclin-induced acute pulmonary vasodilation in primary pulmonary hypertension. Circulation 1982, 66, 334–338. 10.1161/01.CIR.66.2.334. 7046988

[ref5] Provencher S.; John T.; Granton J. T. Current treatment approaches to Pulmonary Arterial Hypertension. Can. J. Cardiol. 2015, 31, 460–477. 10.1016/j.cjca.2014.10.024. 25840096

[ref6] aHellawell J. L.; Bhattacharya S.; Farber H. W. Pharmacokinetic evaluation of treprostinil (oral) for the treatment of pulmonary arterial hypertension. Expert Opin. Drug Metab. Toxicol. 2014, 10, 1445–1453. 10.1517/17425255.2014.958466. 25204984

[ref7] Morrison K.; Ernst R.; Hess P.; Studer R.; Clozel M. Selexipag: a selective prostacyclin receptor agonist that does not affect rat gastric function. J. Pharmacol. Exp. Ther. 2010, 335, 249–255. 10.1124/jpet.110.169748. 20660124

[ref8] Tran T.; Shin Y.-J.; Kramer B.; Choi J.; Zou N.; Vallar P.; Martens P.; Boatman P. D.; Adams J. W.; Ramirez J.; Shi Y.; Morgan M.; Unett D. J.; Chang S.; Shu H.; Tung S.; Semple G. Discovery of a new series of potent prostacyclin receptor agonists with in vivo activity in rat. Bioorg. Med. Chem. Lett. 2015, 25, 1030–1035. 10.1016/j.bmcl.2015.01.024. 25666818

[ref9] Gomez-Arroyo J. G.; Farkas L.; Alhussaini A. A.; Farkas D.; Kraskauskas D.; Voelkel N. F.; Bogaard H. J. The monocrotaline model of pulmonary hypertension in perspective. Am. J. Physiol. Lung Cell Mol. Physiol. 2012, 302, L363–369. 10.1152/ajplung.00212.2011. 21964406

[ref10] Lovering F.; Bikker J.; Humblet C. Escape from flatland: Increasing saturation as an approach to improving clinical success. J. Med. Chem. 2009, 52, 6752–6756. 10.1021/jm901241e. 19827778

[ref11] Ritchie T. J.; Macdonald S. J. F. The impact of aromatic ring count on compound developability – are too many aromatic rings a liability in drug design?. Drug Discovery Today 2009, 14, 1011–1020. 10.1016/j.drudis.2009.07.014. 19729075

[ref12] Nakamura A.; Yamada T.; Asaki T. Synthesis and evaluation of N-acylsulfonamide and N-acylsulfonylurea prodrugs of a prostacyclin receptor agonist. Bioorg. Med. Chem. 2007, 15, 7720–7725. 10.1016/j.bmc.2007.08.052. 17881233

[ref13] Galiè N.; Manes A.; Branzi A. The new clinical trials on pharmacological treatment in pulmonary arterial hypertension. Eur. Respir. J. 2002, 20, 1037–1049. 10.1183/09031936.02.05542002. 12412701

[ref14] Adams J. W.; Ramirez J.; Ortuno D.; Shi Y.; Thomsen W.; Richman J. G.; Morgan M.; Dosa P.; Teegarden B. R.; Al-Shamma H.; Behan D. P.; Connolly D. T. Anti-thrombotic and vascular effects of AR246686, a novel 5-HT2A receptor antagonist. Eur. J. Pharmacol. 2008, 586, 234–243. 10.1016/j.ejphar.2007.11.056. 18358471

[ref15] 95 GPCR profile (MDS Pharma Inc.).

[ref16] Chen G.; Way J.; Armour S.; Watson C.; Queen K.; Jayawickreme C. K.; Chen W. J.; Kenakin T. Use of constitutive G protein-coupled receptor activity for drug discovery. Mol. Pharmacol. 2000, 57, 125–134. 10617687

[ref17] Shen L.; Patel J.; Behan D. B.; Adams J. W.; Clapp L. APD811, a novel and highly selective non-prostanoid IP receptor agonist in smooth muscle cells from patients with pulmonary hypertension. Circulation 2016, 134, A16601.

[ref18] **5c** and all related compounds are highly potent APIs and can cause cutaneous vasodilation following skin exposure. It is also possible that they may cause significant systemic hypotension if swallowed or inhaled. Full PPE precautions were taken and breathing apparatus was used during the conversion of **9** to **5c**.

[ref19] Rudolph G.; Kloeters-Plachky P.; Sauer P.; Stiehl A. Intestinal absorption and biliary secretion of ursodeoxycholic acid and its taurine conjugate. Eur. J. Clin. Invest. 2002, 32, 575–580. 10.1046/j.1365-2362.2002.01030.x. 12190957

