# Supplementary material for: Discovery of 2-(((1r,4r)-4-(((4-Chlorophenyl)(phenyl)carbamoyl)oxy)methyl)cyclohexyl)methoxy)acetate (Ralinepag): An Orally Active Prostacyclin Receptor Agonist for the Treatment of Pulmonary Arterial Hypertension
Source: J Med Chem. 2017 Jan 10;60(3):913–27. doi: 10.1021/acs.jmedchem.6b00871 (PMC11602115; doi:10.1021/acs.jmedchem.6b00871)
Supplement: Supplementary file 1 — jm6b00871_si_001.pdf [file jm6b00871_si_001.pdf]

Discovery of 2-(((1*r*,4*r*)-4-(((4-chlorophenyl)(phenyl)carbamoyl)oxy)methyl) cyclohexyl)methoxy)acetate (Ralinepag) : A Orally Active Prostacyclin Receptor Agonist for the Treatment of Pulmonary Arterial Hypertension

Thuy-Anh Tran, Bryan Kramer, Young-Jun Shin, Pureza Vallar, P. Douglas Boatman, Ning Zou, Carleton R. Sage, Tawfik Gharbaoui, Ashwin Krishnan, Biman Pal, Sagar Shakya, Antonio Garrido Montalban, John W. Adams, Juan Ramirez, Dominic P. Behan, Anna Shifrina, Anthony Blackburn, Tina Leakakos, Yunqing Shi, Michael Morgan, Abu Sadeque, Weichao Chen, David J. Unett, Steve Chang, Hsin-Hui Shu, Shiu-Feng Tung and Graeme Semple*

Arena Pharmaceuticals, 6154 Nancy Ridge Drive, San Diego, CA, 92121, USA.

Supporting Information

Table of Contents

S2: Additional In Vitro Assays

S3: Additional data from the in vitro and vivo evaluation of **5c**

S4: PK Profile of **5c** (sodium salt) in cynomolgus monkey

S5: SC-TGA and Adsorption-Desorption profiles of **5c** and **5g**

S7: Broad screening (binding) profile of **5c**

Additional In Vitro Assays

To determine the potency and efficacy of compounds in cAMP assays in the absence of receptor reserve effects, the cDNA sequence of the human IP receptor was sub-cloned into an expression plasmid containing a ubiquitin promoter in place of the more commonly used CMV promoter. The ubiquitin promoter yields a greatly reduced mRNA copy number than the CMV promoter and helps reduce the level of receptor expression after transfection into a suitable cell line. This plasmid was transiently transfected into CHO cells using lipofectamine, following standard protocols. The quantity of transfected plasmid DNA was titrated down from 16 μg to 1 ng per transfection using empty vector as carrier DNA. Transfected cells were used in standard HTRF cAMP assays, as described in the Experimental section, 48 hours post transfection. Data were collected from cells that provided the minimum detectable cAMP response (typically cells transfected with 1 – 10 ng of plasmid).

Compounds were also evaluated in HTRF cAMP assays using primary human pulmonary arterial smooth muscle cells (Cell Applications, Inc., #352K-05a, or ThermoFisher/Gibco, #C0095C) using the same basic assay protocol outlined in the Experimental section. Cells were cultured as recommended by the suppliers, used at low passage and seeded into 384-well assay plates at a density of 700-900 cell per well

Table sup1: Functional evaluation of **5c** and comparator compounds in recombinant IP receptor cAMP assays following elimination of receptor reserve and in primary human pulmonary arterial smooth muscle cells.

**Compound**	**Test System**	**EC_{50} (nM) [95% CI] (n)**	**Efficacy (% vs. Iloprost) [95% CI]**
Iloprost	Recombinant IP receptor cAMP	3.3 [1.7, 6.4] (9)	100
	Primary human PASMC cAMP	2.9 [1.6, 5.3] (8)	100
**2**	Recombinant IP receptor cAMP	151 [112, 203] (9)	48 [43, 53]
	Primary human PASMC cAMP	184 [136, 248] (8)	41 [33, 49]
**5c**	Recombinant IP receptor cAMP	24 [20, 29] (9)	67 [64, 71]
	Primary human PASMC cAMP	24 [31, 40] (8)	65 [55, 75]

Table Sup2: Data with some comparator compounds in functional assays.

Compound	DP1 EC_{50}, µM (% I.A)	EP1 EC_{50}, µM (% I.A)	EP2 EC_{50}, µM (% I.A)	EP3v6 EC_{50}, µM (% I.A)	EP4 EC_{50}, µM (% I.A)
PGD_2	0.0013 (36) n = 36	> 10	> 10	3.1 (98) n = 3	> 10
PGE_2	0.52 (28) n = 28	0.051 (92) n = 4	0.0055 (84) n = 39	0.0001(100) n = 9	*n.d.*
BW245C	0.0008 (98) n = 26	*n.d.*	0.79 (73) n = 13	*n.d.*	> 10
Iloprost	0.147 (42) n = 3	0.047 (48) n = 2	0.144 (110) n = 8	0.28 (68) n = 6	0.41 (96) n = 6
**2**	0.154 (80) n = 2	*n.d.*	> 10	> 10	> 10
**5c**	0.850 (52) n = 52	> 10	> 10	0.230 (98) n = 51	> 10

All assays were cAMP HTRF agonist assays (G_s) except for EP3v6 which was a melanophore dispersion assay (G_i).

n.d. = not determined
n ≥ 3 for all compounds with EC_{50} > 10µM

***Additional data from the in vivo evaluation of* 5c**

Effect of high dose **5c** increase in pulmonary arterial wall thickness. As this was a time consuming assay, only 5 animals were selected at random from each of group for these measurements. As the 10mg/kg group did not show a statistically significant effect on hypertrophy, no tissue from that group was tested.

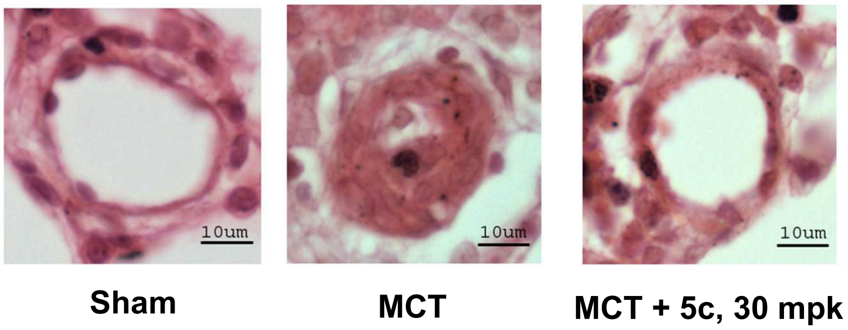

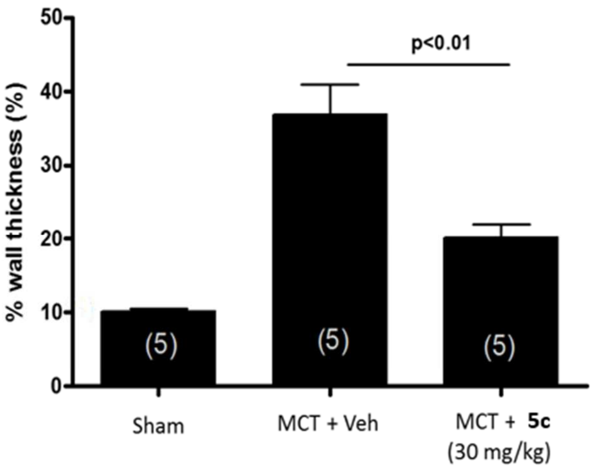

PK Profile of 5c (sodium salt) in cynomolgus monkey

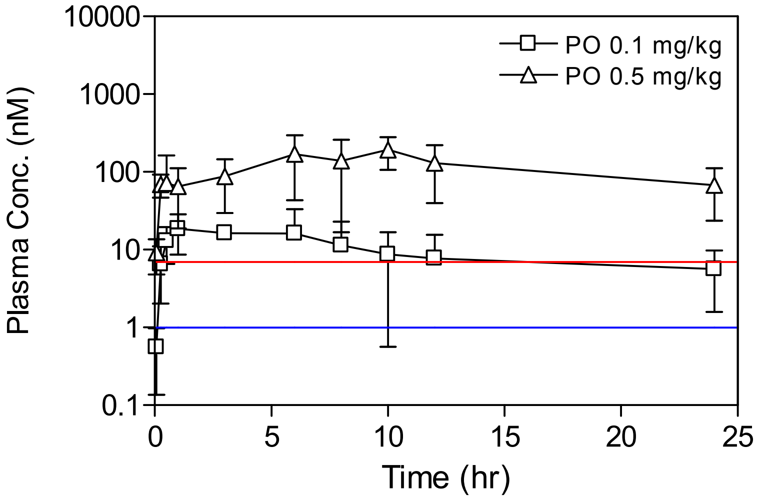

Dose (mg/kg)	Tmax (hr)	$T_{1/2}$ (hr)	C_{max} (nM)	C_{trough} (nM)	C_{max}/C_{trough}
0.1	2.67	17.5	28.0	5.67	**5**
0.5	4.17	33.5	215	67.8	**3**

DSC-TGA and Adsorption-Desorption profiles of 5c and 5g (Sodium salts)

5c

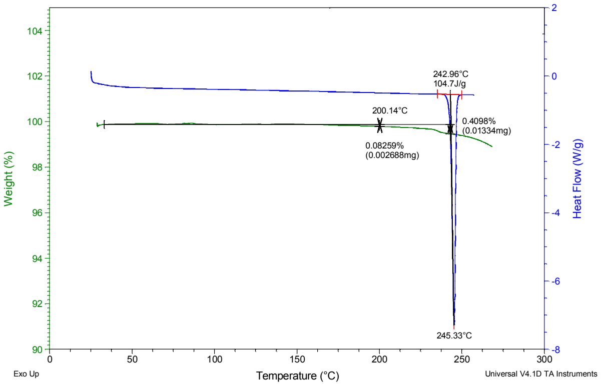

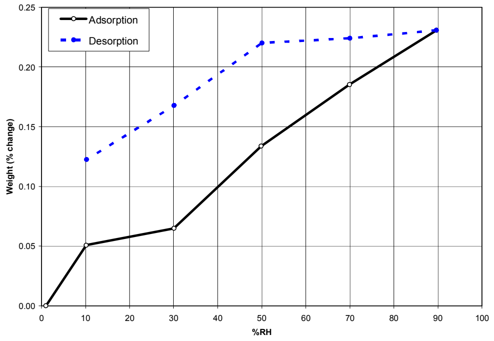

5g

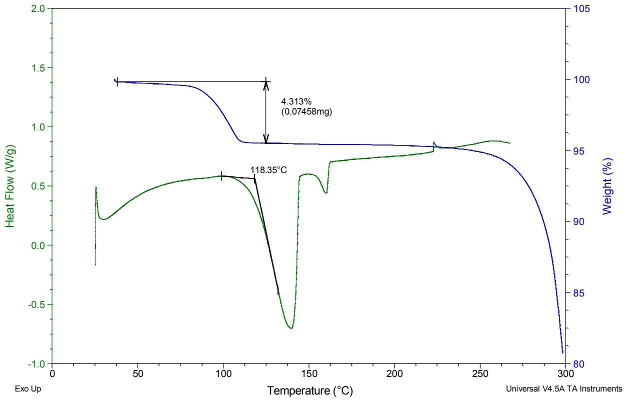

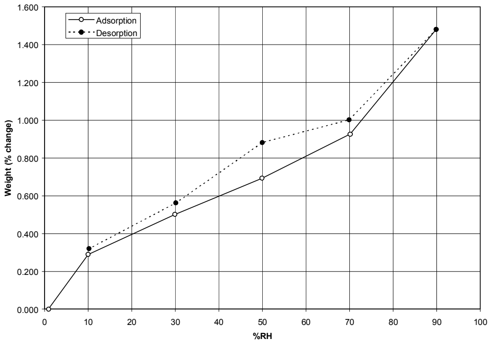

DSC-TGA and Adsorption-Desorption profile of 5c (APD811) Free acid

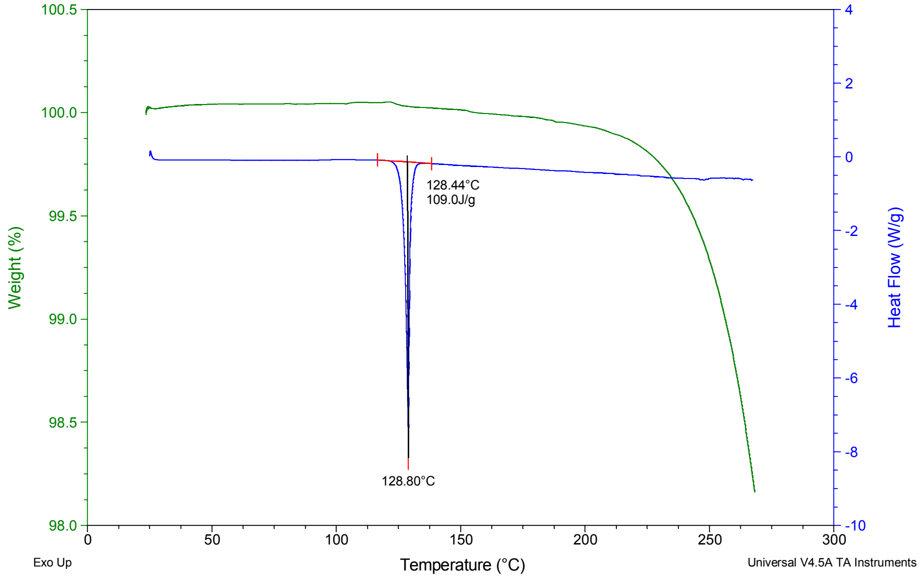

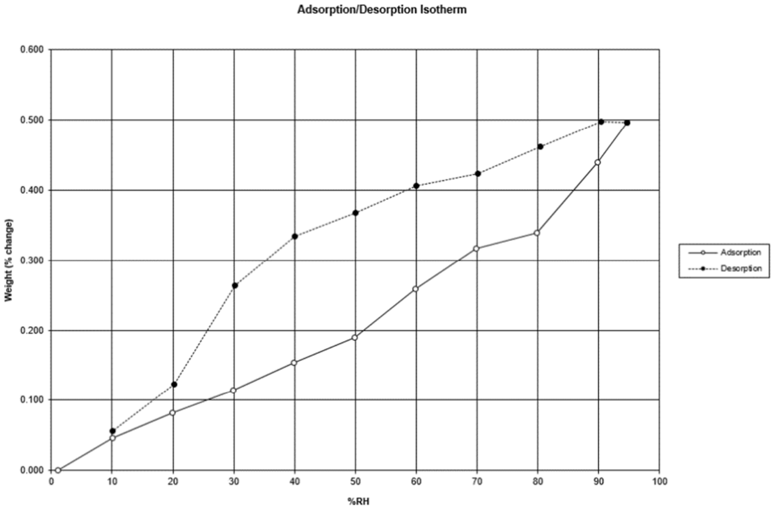

Broad screening profile of 5c (MDS Pharma)

Cat. #	TARGET	BATCH*	SPP.	n=	CONC.	†% INHIBITION (%)	IC_{50}	K_i	n_H	R
200510	Adenosine A_1	216976	hum	2	10 μM	14				
200610	Adenosine A_{2A}	216977	hum	2	10 μM	4				
200720	Adenosine A_3	217106	hum	2	10 μM	-1				
203400	Adrenergic α_{1D}	217023	hum	2	10 μM	1				
203620	Adrenergic α_{2A}	217024	hum	2	10 μM	9				
203710	Adrenergic α_{2B}	217025	hum	2	10 μM	12				
203800	Adrenergic α_{2C}	217026	hum	2	10 μM	5				
204010	Adrenergic β_1	217142	hum	2	10 μM	7				
204110	Adrenergic β_2	217143	hum	2	10 μM	-10				
204200	Adrenergic β_3	217144	hum	2	10 μM	8				
204460	Adrenomedullin AM_1	216980	hum	2	10 μM	-5				
204470	Adrenomedullin AM_2	216981	hum	2	10 μM	-4				
210020	Angiotensin AT_1	217109	hum	2	10 μM	16				
210110	Angiotensin AT_2	217110	hum	2	10 μM	-2				
211600	Bombesin BB1	216962	hum	2	10 μM	2				
211700	Bombesin BB2	216963	hum	2	10 μM	-4				
211800	Bombesin BB3	216964	hum	2	10 μM	-1				
212510	Bradykinin B_1	216982	hum	2	10 μM	-17				
212610	Bradykinin B_2	216983	hum	2	10 μM	-6				
213610	Calcitonin	217099	hum	2	10 μM	2				
214010	Calcitonin Gene-Related Peptide $CGRP_1$	216965	hum	2	10 μM	9				
217020	Cannabinoid CB_1	217100	hum	2	10 μM	9				
217100	Cannabinoid CB_2	217101	hum	2	10 μM	-21				
217500	Chemokine CCR1	217073	hum	2	10 μM	0				
217550	Chemokine CCR2B	217074	hum	2	10 μM	3				
217650	Chemokine CCR4	217075	hum	2	10 μM	-1				
217700	Chemokine CCR5	217076	hum	2	10 μM	-3				
244600	Chemokine CX3CR1	217083	hum	2	10 μM	-3				
244300	Chemokine CXCR1/2 (IL-8, Non-Selective)	217081	hum	2	10 μM	4				
244500	Chemokine CXCR2 (IL-$8R_B$)	217082	hum	2	10 μM	-3				
218120	Cholecystokinin CCK_2 (CCK_B)	217273	hum	2	10 μM	-4				

Cat. #	TARGET	BATCH*	SPP.	n=	CONC.	† % INHIBITION %	IC_{50}	K_I	n_H	R
219150	Corticotropin Releasing Factor CRF_1	217306	hum	2	10 µM	14				
219500	Dopamine D_1	217422	hum	2	10 µM	1				
219600	Dopamine D_{2L}	217116	hum	2	10 µM	-4				
219700	Dopamine D_{2S}	217118	hum	2	10 µM	7				
219800	Dopamine D_3	217425	hum	2	10 µM	-1				
219900	Dopamine $D_{4.2}$	217077	hum	2	10 µM	-13				
220000	Dopamine $D_{4.4}$	217120	hum	2	10 µM	-1				
220100	Dopamine $D_{4.7}$	217122	hum	2	10 µM	-8				
220200	Dopamine D_5	217044	hum	2	10 µM	-3				
224010	Endothelin ET_A	217078	hum	2	10 µM	-8				
224110	Endothelin ET_B	217079	hum	2	10 µM	-1				
226300	G Protein-Coupled Receptor GPR103	216966	hum	2	10 µM	1				
226230	G Protein-Coupled Receptor GPR8	217146	hum	2	10 µM	20				
228610	$GABA_{B1A}$	217270	hum	2	10 µM	18				
228710	$GABA_{B1B}$	217271	hum	2	10 µM	19				
231510	Galanin GAL1	216988	hum	2	10 µM	-2				
231600	Galanin GAL2	216989	hum	2	10 µM	1				
239300	Growth Hormone Secretagogue (GHS, Ghrelin)	217284	hum	2	10 µM	-2				
239610	Histamine H_1	217029	hum	2	10 µM	-5				
239710	Histamine H_2	217030	hum	2	10 µM	-2				
239810	Histamine H_3	217031	hum	2	10 µM	-15				
239900	Histamine H_4	217032	hum	2	10 µM	-5				
250510	Leukotriene, BLT (LTB_4)	217282	hum	2	10 µM	-16				
250460	Leukotriene, Cysteinyl $CysLT_1$	217312	hum	2	10 µM	8				
250480	Leukotriene, Cysteinyl $CysLT_2$	217313	hum	2	10 µM	10				
251100	Melanocortin MC_1	217047	hum	2	10 µM	5				
251300	Melanocortin MC_3	217048	hum	2	10 µM	5				
251350	Melanocortin MC_4	217338	hum	2	10 µM	2				
251400	Melanocortin MC_5	217049	hum	2	10 µM	-13				
251600	Melatonin MT_1	217266	hum	2	10 µM	2				

Cat. #	TARGET	BATCH*	SPP.	n=	CONC.	†% INHIBITION %	IC$_{50}$	K$_I$	n$_H$	R
251700	Melatonin MT_2	217805	hum	2	10 µM	11				
252200	Motilin	217084	hum	2	10 µM	2				
252610	Muscarinic M_1	217036	hum	2	10 µM	7				
252710	Muscarinic M_2	217034	hum	2	10 µM	-2				
252810	Muscarinic M_3	217035	hum	2	10 µM	-12				
252910	Muscarinic M_4	217441	hum	2	10 µM	1				
253010	Muscarinic M_5	217442	hum	2	10 µM	2				
226100	N-Formyl Peptide Receptor FPR1	217045	hum	2	10 µM	-2				
226200	N-Formyl Peptide Receptor-Like FPRL1	217046	hum	2	10 µM	12				
256100	Neuromedin U NMU_1	217052	hum	2	10 µM	5				
256200	Neuromedin U NMU_2	217053	hum	2	10 µM	2				
257010	Neuropeptide Y Y_1	217379	hum	2	10 µM	6				
257110	Neuropeptide Y Y_2	217085	hum	2	10 µM	-4				
258010	Neurotensin NT_1	217264	hum	2	10 µM	1				
260110	Opiate δ (OP1, DOP)	216991	hum	2	10 µM	8				
260210	Opiate κ (OP2, KOP)	216993	hum	2	10 µM	-5				
260410	Opiate µ (OP3, MOP)	216994	hum	2	10 µM	0				
260600	Orphanin ORL_1	217260	hum	2	10 µM	10				
265010	Platelet Activating Factor (PAF)	217261	hum	2	10 µM	0				
268030	Prostanoid CRTH2	217318	hum	2	10 µM	19				
◆ 268050	Prostanoid DP	216971	hum	2	10 µM	80				
◆ 268200	Prostanoid EP_2	216972	hum	2	10 µM	91				
◆ 268410	Prostanoid EP_4	216973	hum	2	10 µM	83				
285510	Prostanoid, Thromboxane A_2 (TP)	217107	hum	2	10 µM	46				
271110	Serotonin (5-Hydroxytryptamine) $5\text{-}HT_{1A}$	217131	hum	2	10 µM	0				
271700	Serotonin (5-Hydroxytryptamine) $5\text{-}HT_{2B}$	217135	hum	2	10 µM	-14				
271800	Serotonin (5-Hydroxytryptamine) $5\text{-}HT_{2C}$	217136	hum	2	10 µM	-19				
272100	Serotonin (5-Hydroxytryptamine) $5\text{-}HT_{5A}$	217139	hum	2	10 µM	-8				

Cat. #	TARGET	BATCH*	SPP.	n=	CONC.	%	†% INHIBITION (-100 -50 0 50 100)	IC_{50}	K_i	n_H	R
272200	Serotonin (5-Hydroxytryptamine) 5-HT_6	217140	hum	2	10 μM	-5					
282510	Somatostatin sst1	217253	hum	2	10 μM	4					
282700	Somatostatin sst2	217254	hum	2	10 μM	-9					
282530	Somatostatin sst3	217255	hum	2	10 μM	-1					
282900	Somatostatin sst4	217256	hum	2	10 μM	19					
283000	Somatostatin sst5	217257	hum	2	10 μM	14					
255510	Tachykinin NK_1	217277	hum	2	10 μM	-19					
255600	Tachykinin NK_2	217279	hum	2	10 μM	8					
255710	Tachykinin NK_3	217604	hum	2	10 μM	4					
286700	Urotensin II	217258	hum	2	10 μM	-3					
287010	Vasoactive Intestinal Peptide VIP_1	217088	hum	2	10 μM	0					
287530	Vasopressin V_{1A}	217089	hum	2	10 μM	1					
287560	Vasopressin V_{1B}	217090	hum	2	10 μM	6					
287610	Vasopressin V_2	217091	hum	2	10 μM	-19					
